# The effects of Phycocyanobilin on experimental arthritis involve the reduction in nociception and synovial neutrophil infiltration, inhibition of cytokine production, and modulation of the neuronal proteome

**DOI:** 10.3389/fimmu.2023.1227268

**Published:** 2023-10-23

**Authors:** Javier Marín-Prida, Arielis Rodríguez-Ulloa, Vladimir Besada, Alexey Llopiz-Arzuaga, Nathália Vieira Batista, Ignacio Hernández-González, Nancy Pavón-Fuentes, Érica Leandro Marciano Vieira, Viviana Falcón-Cama, Emilio F. Acosta, Gillian Martínez-Donato, Majel Cervantes-Llanos, Dai Lingfeng, Luis J. González, Julio Raúl Fernández-Massó, Gerardo Guillén-Nieto, Eduardo Pentón-Arias, Flávio Almeida Amaral, Mauro Martins Teixeira, Giselle Pentón-Rol

**Affiliations:** ^1^ Center for Research and Biological Evaluations, Institute of Pharmacy and Food, University of Havana, Havana, Cuba; ^2^ Division of Biomedical Research, Center for Genetic Engineering and Biotechnology, Havana, Cuba; ^3^ China-Cuba Biotechnology Joint Innovation Center (CCBJIC), Yongzhou Zhong Gu Biotechnology Co. Ltd, Yongzhou, China; ^4^ Department of Cellular Engineering and Biocatalysis , Institute of Biotechnology, National Autonomous University of Mexico (UNAM), Cuernavaca, Mexico; ^5^ Laboratory of Immunopharmacology, Department of Biochemistry and Immunology, Instituto de Ciências Biológicas, Universidade Federal de Minas Gerais, Belo Horizonte, Minas Gerais, Brazil; ^6^ Department of Non-Clinical Research, Isotopes Center, San José de Las Lajas, Mayabeque, Cuba; ^7^ Immunochemical Department, International Center for Neurological Restoration (CIREN), Havana, Cuba; ^8^ Translational Psychoneuroimmunology Group, School of Medicine, Federal University of Minas Gerais (UFMG), Belo Horizonte, Brazil; ^9^ Departments of Physiological or Morphological Sciences, Latin American School of Medicine (ELAM), Havana, Cuba; ^10^ Department of Characterization, Center for Advanced Studies of Cuba, Havana, Cuba

**Keywords:** Phycocyanobilin, rheumatoid arthritis, hypernociception, glutamatergic transmission, proteome, neutrophils, inflammation, C-Phycocyanin

## Abstract

**Introduction:**

The antinociceptive and pharmacological activities of C-Phycocyanin (C-PC) and Phycocyanobilin (PCB) in the context of inflammatory arthritis remain unexplored so far. In the present study, we aimed to assess the protective actions of these compounds in an experimental mice model that replicates key aspects of human rheumatoid arthritis.

**Methods:**

Antigen-induced arthritis (AIA) was established by intradermal injection of methylated bovine serum albumin in C57BL/6 mice, and one hour before the antigen challenge, either C-PC (2, 4, or 8 mg/kg) or PCB (0.1 or 1 mg/kg) were administered intraperitoneally. Proteome profiling was also conducted on glutamate-exposed SH-SY5Y neuronal cells to evaluate the PCB impact on this key signaling pathway associated with nociceptive neuronal sensitization.

**Results and discussion:**

C-PC and PCB notably ameliorated hypernociception, synovial neutrophil infiltration, myeloperoxidase activity, and the periarticular cytokine concentration of IFN-γ, TNF-α, IL-17A, and IL-4 dose-dependently in AIA mice. In addition, 1 mg/kg PCB downregulated the gene expression for T-bet, RORγ, and IFN-γ in the popliteal lymph nodes, accompanied by a significant reduction in the pathological arthritic index of AIA mice. Noteworthy, neuronal proteome analysis revealed that PCB modulated biological processes such as pain, inflammation, and glutamatergic transmission, all of which are involved in arthritic pathology.

**Conclusions:**

These findings demonstrate the remarkable efficacy of PCB in alleviating the nociception and inflammation in the AIA mice model and shed new light on mechanisms underlying the PCB modulation of the neuronal proteome. This research work opens a new avenue to explore the translational potential of PCB in developing a therapeutic strategy for inflammation and pain in rheumatoid arthritis.

## 1 Introduction

Rheumatoid arthritis (RA) stands as a persistent autoimmune disease, marked by widespread synovitis and, at times, by relentless bone deterioration (1). The ensuing joint abnormalities, which include rigidity and deformity, precipitate a distressing setback of mobility in RA-afflicted individuals and eventually lead to varying degrees of bone decomposition, harm to ligaments and tendons, and skeletal muscle weakening (2).

The initiation and extension of chronic RA inflammation involves both adaptive and innate immune cells. Neutrophils have been identified as the predominant leukocyte in the joints of individuals with active RA (3). Their role in the disease’s pathogenesis includes tissue damage and the discharge of proinflammatory cytokines as well as an important crosstalk with other immune cells such as T cells and dendritic cells (DCs) (4–6). Animal models of arthritis have provided more direct evidence of neutrophil involvement in this disease. In the K/BxN mouse RA model, neutrophil depletion led to the complete reversion of the joint inflammation, showing no signs of swelling either in the forefeet or the ankle joints (7). Likewise, chemokines commonly found in rheumatoid synovial fluid drew neutrophils into the affected joints in an arthritis mice model induced by collagen, while the neutrophil depletion also completely prevented the disease development in this model (8).

Furthermore, impairment of the resolution of inflammation acquires a prominent role in perpetuating clinical dysfunction in chronic diseases such as RA. It has been noted that a persistent failure in neutrophil death is correlated with an increased severity of experimental arthritis in mice (9). Even when neutrophils die, their disposal is dependent on the expression of “eat-me” signals that trigger the engulfing activity of phagocytes, a process called efferocytosis, which may also fail during RA (10). Therefore, considering the diverse roles of neutrophils uncovered in the development of RA, it comes as no surprise that they have become crucial focal points for potential novel disease-modifying treatments.

The prevailing symptom of RA is pain, as confirmed by noteworthy data showing that 97% of individuals with early RA experience pain, and this serves as the primary cause for their initial consultation with healthcare practitioners (11). The onset of pain precedes the visible signs of RA (12, 13), leading to psychological affliction and disturbances in sleep patterns (14). Furthermore, pain emerges as a pivotal factor that influences crucial aspects of daily life. Even when the pain intensity is mild, it can significantly impede regular activities (15). The joint’s synovium and capsule primarily house the peripheral afferent fibers stemming from the dorsal root ganglion (DRG). Within these regions, a considerable population of primary Aα and Aβ sensory neurons are engaged in mechanosensation, while Aδ and C fibers are responsible for nociception (16). Additionally, sensory periphery nerves are also distributed throughout the joint capsule, lateral area of the meniscus, subchondral bone, ligaments, tendon sheaths, and muscles. All these areas also contribute significantly to the emergence of arthritic pain by exposing their innervating nerves to sensitizing factors secondary to the arthritic progressive tissue erosion (17). Notably, synovitis within the joint stands as a key pathophysiological mechanism of RA pain generation, directly engaging and sensitizing the afferent nerves of the periphery through a range of soluble mediators, including bradykinin, prostaglandins, cytokines, and the main excitatory neurotransmitter glutamate (18, 19).

Indeed, glutamatergic signaling in the joints has emerged as a major factor in the pathophysiology of arthritic pain. Numerous studies have consistently reported a significant increase in glutamate concentration in the synovial fluid of humans with RA (20, 21) and in animal models of this disease (22). In one study, a single intra-articular injection of a glutamate receptor antagonist successfully inhibited allodynia, a type of pain that arises from a typically non-painful stimulus, in rats with complete Freund’s adjuvant-induced arthritis (23). The accumulation of glutamate in the arthritic joint is believed to originate from the peripheral dorsal root ganglion (DRG) nerve terminals due to a marked increase in the production of glutaminase, the primary glutamate synthetic enzyme in neurons (24). This excess glutamate is subsequently released into the extracellular space, leading to an autocrine or paracrine sensitization process. Additional evidence supporting this conclusion comes from studies showing that inhibiting glutaminase peripherally has potent analgesic effects in rats with carrageenan-induced paw inflammation (25).

In parallel to this peripheral sensitization, the mechanisms that regulate pain in the central nervous system are also critically dependent on the sensitization of neurons present in the dorsal horn of the spinal cord, which receive input from the DRG Aδ and C fibers. This central sensitization, caused by the hyperexcitability of spinal neurons, accompanied by a shortage or augmentation of descending inhibitory or facilitatory pathways, respectively, leads to the amplification of the receptive field and an increase in pain sensitivity (26). Distinctive phases occurring in this spinal sensitization of RA pain have been postulated. In the acute phase of the disease, this phenomenon is dictated by the enhanced release of glutamate from the DRG afferent presynaptic endings, which acts on its ionotropic (NMDA, AMPA, kainite) and metabotropic receptors present in the postsynaptic neurons of the spinal dorsal horn (27). In the long-lasting stage of RA, a marked influence of microglia and astrocyte activity on synaptic processing has been documented in diverse chronic models of this malady (28).

Based on these premises, it is reasonable to investigate how neurons respond to excitatory glutamatergic stimulation to predict the possible mediators of inflammatory pain processing. Proteomics studies have proven to be a valuable tool in identifying important cellular markers and pathways of neuronal responses and translatability at a mechanistic level (29). Among those extensively studied cellular models is the human SH-SY5Y neuronal cell line, known for its predictability in assessing neuronal glutamate receptors excitability (30) and responses to inflammatory stimuli such as lipopolysaccharide (31) or cytokines (32). Utilizing this model provides an appropriate experimental framework to detect distinct changes in proteomic profiles under glutamatergic stimulation, potentially leading to new mechanistic hypotheses.

The primary objective of RA therapy is to attain disease remission by easing its symptoms and enhancing the overall quality of life. RA treatment currently involves five main drug classes: pain relievers, non-steroidal anti-inflammatory drugs, glucocorticoids, biologic disease-modifying antirheumatic drugs (bDMARDs), and non-biologic or synthetic DMARDs (33). Despite their usefulness, these treatment approaches often come with undesirable side effects, and approximately 20-40% of patients with RA do not exhibit a positive clinical response to these therapies (34). As a result, there is a pressing need for innovative treatment alternatives to achieve this unmet need in RA treatments.

C-Phycocyanin (C-PC) is the main protein composing the phycobilisomes of *Spirulina platensis* microalgae. This phycobiliprotein is constituted by the α and β subunits, polypeptides with molecular weights of 17.6 and 18 kDa, respectively, which contain as a prosthetic group, a tetrapyrrolic ring structure named Phycocyanobilin (PCB) that is linked by thioether bonds to cysteines 84 (α chain), 82 and 153 (β chain) (35). This compound functions as the chromophore of the protein complex for energy transduction in the cyanobacteria´s phycobilisomes, and it is associated with the antioxidant (36) and immunomodulatory (37) activities of C-PC. The beneficial properties of *Spirulina platensis* extracts, the natural source of C-PC/PCB, have been studied in several animal models of arthritis (38). By exerting a combination of antioxidant, antiangiogenic, and anti-inflammatory actions, the raw preparations of *Spirulina platensis* have shown promising evidence for alleviating arthritic injuries (39–41). However, to the best of our knowledge, there is no previous report describing the antinociceptive and inflammopharmacological activities of PCB in the context of inflammatory arthritis.

Considering their properties, here we provide evidence that PCB, either administered in pure form or released *in vivo* from its prodrug, the biliprotein C-PC, has protective actions in an experimental setting that mimics several hallmarks of the human disease RA in mice, with emphasis on arthritis-associated nociception and inflammation. This hypothesis was demonstrated through observations that highlight this therapy’s potential to effectively cope with functional, immunological, and pathological arthritic injuries. Furthermore, we aimed to assess the effects of PCB on glutamate-induced proteome changes in SH-SY5Y neurons, seeking novel mechanistic insights that may explain the counteractive actions of this molecule against arthritis nociception.

## 2 Materials and methods

### 2.1 Reagents

Sigma-Aldrich (St. Louis, USA) was the commercial supplier of the reagents for most experiments, except in those accordingly indicated. The raw material of *Spirulina platensis* was a kind gift from Genix (Labiofam, Havana, Cuba). The extraction of C-PC from this biomass followed the procedures already standardized and published by our group, which are confirmed by an aqueous two-phase separation system (42). The final C-PC purity solution was higher than 4 (analytical grade) and this stock was kept refrigerated at 4°C during the duration of the experiments. The working dilutions of C-PC were prepared with sterile phosphate buffer saline (PBS) pH 7.4 immediately before its administration to mice. PCB was commercially obtained in powder (Cat. No. SC-396921, Santa Cruz Biotechnology, Inc., Dallas, USA), diluted with sterile PBS pH 7.4 at 5 mg/mL, and stocked in frozen aliquots (-20°C). Immediately before the administration, the PCB solutions (light-protected) were diluted from the stock according to the scheduled dose.

### 2.2 Laboratory mice

Male C57BL/6 mice (6-8 weeks old) provided by the Federal University of Minas Gerais (UFMG) (43), Belo Horizonte, Brazil, were maintained in the animal rooms of the Immunopharmacology Laboratory, Department of Biochemistry and Immunology at UFMG. Animals received standard food and filtered water *ad libitum* with temperature and humidity-controlled conditions. All procedures involving animal care and handling were approved by the UFMG ethics committee (CEUA UFMG:165/2009).

### 2.3 Antigen-induced arthritis in mice and treatment schedules

AIA was induced following a previously described procedure (43). Briefly, mice were intraperitoneally anesthetized with a mixture of 100 mg/kg of ketamine and 10 mg/kg of xylazine (44) and then immunized by an intradermal shot at the tail base of an emulsion containing 500 µg of methylated bovine serum albumin (mBSA; Sigma) and 100 µL of saline plus Freund’s complete adjuvant (CFA; Sigma) at 1:1 ratio (v:v). After two weeks, the challenge with 10 µg of mBSA (in 10 µL sterile saline) was performed by injecting it intra-articularly in the right knee joint of anesthetized mice. At the appropriate time points, euthanasia of the mice was carried out as permitted according to Annex IV of Directive 2010/63/EU, with an anesthetic overdose (180 mg/kg of ketamine and 24 mg/kg of xylazine, intraperitoneally).

Three separate experiments and different outcome assessments were performed. In the first experiment, five groups of mice (n=5-6 each one) were separated at random and divided into groups composed of the control mice receiving an injection of 10 μL sterile saline in the same joint and mice with AIA treated intraperitoneally either with vehicle (PBS pH 7.4) or with C-PC at 2, 4, or 8 mg/kg one hour before the antigen challenge. In the second experiment, the same treatment schedule and route of administration were used, and the mice were allocated at random into four groups (n=6 each one) made up of, in addition to the control, the diseased animals treated with increasing doses of PCB (0.1 or 1 mg/kg), or with PBS pH 7.4 (defined as the vehicle). In the first two experiments, the hypernociception (a pain index measured in the affected right paw), the myeloperoxidase (MPO) activity, and the neutrophil infiltration in the affected knee cavity were determined following previously described procedures (45).

Finally, the third experiment used a similar design to the second. It included the control, the AIA + vehicle, and the AIA + PCB 1 mg/kg groups (n=4-6 each one) but the experiment aimed to perform a histopathological evaluation of the diseased knee.

### 2.4 Evaluation of hypernociception

An electronic pressure device equipped with a polypropylene tip (4.15 mm^2^) transducer was utilized (Insight Instruments, Ribeirão Preto, Sao Paulo, Brazil). Prior to the study, mice were subjected to a 30-min room adaptation when housed in acrylic cages (12 x 10 x 17 cm high) with a wired floor. Mechanical stimulation was realized while the mice were completely calm, by applying a force with the transducer tip to the central field of the affected paw. Consequently, the bending of the femorotibial joint was produced, accompanied by the retraction of the stimulated paw. The device registered the force intensity when the paw retreat was completed, and the results were expressed as the change in withdrawal threshold (in grams) (46).

### 2.5 Determination of MPO activity and CXCL1 levels

The assessment of the MPO activity and the CXCL1 protein levels was performed in the periarticular tissue homogenate coming from the right knee joint and processed by Ultra-Turrax (Ika, Minas Gerais, Brazil). The MPO activity was evaluated as described (47), by the enzymatic reaction of 25 µL of sample in the presence of 25 µL of 3,3’-5,5’-tetramethylbenzidine (TMB) at 1.6 mM as the color reagent, followed by the spectrophotometric measurement of the reaction product at 450 nm. The absorbance values were interpolated in a standard curve made with a simultaneous assay on 5% casein peritoneal-induced neutrophils, and results were expressed as relative arbitrary units.

CXCL1 was measured by an ELISA kit as indicated by the supplier instructions manual (Duo-Set kits, R&D Systems, Minneapolis, MN, USA).

### 2.6 Intra-articular neutrophil quantification

The cavity of the right knee was cleansed with sterile PBS (two times with 5 µL), added to 90 µL sterile PBS, and stored on ice. The total quantity of leukocytes was immediately counted with a Neubauer chamber when the samples (10 µL) were stained with Turk’s solution. The remaining samples were mounted on slides through a cytospin instrument (Shandon III; Thermo Shandon, Frankfurt, Germany). Differential leukocyte assessment in these slides was performed with a May-Grünwald-Giemsa staining following standard morphologic parameters (48).

### 2.7 Real-time PCR

Total RNA was extracted from popliteal lymph nodes (LNs) with TRIzol reagent (Invitrogen, Rockville, MD) (49). All qPCR assays were done in triplicate with gene-specific primers (**Supplementary Table S1**), which were used at 300 nM (OriGene Technologies, USA). The reaction products were detected with Fast SYBR Green PCR Master Mix in the Applied Biosystems 7900HT Fast Real-Time PCR System (Applied Biosystems, Foster City, CA, USA). Bi-distilled water was used as a negative control for all assays, either for the target or for the housekeeping genes. The results were calculated following the 2-ΔΔCt method (50).

### 2.8 Cytometric Bead Array

After careful dissection of the periarticular tissues, these were homogenized with Ultra-Turrax (Ika, Minas Gerais, Brazil) in the presence of a protease-inhibitor mix in PBS pH 7.4. Supernatants were obtained by centrifugation at 13,000g for 10 min at 4 °C and frozen-stored (-70 °C) until needed for the CBA assay. Four cytokines were evaluated (TNF-α, IFN-γ, IL-17A, and IL-4) following the kit’s protocol (BD™ CBA Mouse Inflammation Kit, BD Biosciences, San Diego, CA), and quantified on a FACS Calibur flow cytometer (Becton Dickinson, San Jose, CA).

### 2.9 Histopathological analysis

The right knee joints were collected for histological evaluation. After the fixation step in 10% buffered formalin (pH 7.4), a 30-day decalcification phase was done by incubating the samples in 14% EDTA pH 7.2 at 20-25 °C prior to paraffin embedding, cutting in sections, and staining with 5 μM hematoxylin/eosin. A pathology specialist blindly analyzed two sections/knee joints with a light microscope and assigned a score to each of the following parameters: hyperplasia of the synovium, immune cell infiltration, and bone erosion. The arthritis index was calculated by summing the score of each of these parameters, ranging from 0 to 8, with a higher index indicating an increased injury (51). Representative images for each experimental group were taken with a microscope-coupled digital camera and processed with Image J software (National Institutes of Health, Bethesda, MD).

### 2.10 Isotopic labeling

The radioactive labeling was accomplished with ^125^I using the Iodogen method (52). Briefly, an Iodogen coated 0.5 mL microtube (Eppendorf, USA) was placed in 1 M phosphate buffer (pH 7.0), using 37 MBq [^125^I]-NaI (Isotop, Hungary) in 1 M NaOH and gently stirred for 5 min. Afterward, the labeling was done through the addition of 100 μg of C-PC dissolved in 100 μL of purified water. Then, the radiolabel was continued for an additional time of 20 min. The labeled C-PC was separated from the reaction medium in a Sephadex G25 column (GE Healthcare, USA) previously equilibrated in 1 M phosphate buffer (pH 7.0). Finally, the blue fractions with radiochemical purities of at least 90% were pooled and stored at 4°C until use. Blood samples (70 μL) were collected from the retro-orbital plexus by means of a heparinized capillary in anesthetized rats at 1, 15, and 30 min after the administration of 5 mg/kg C-PC. All samples were centrifuged at 10,000 rpm for 5 min. The 20µL plasma aliquot was added to 0.5 mL of 0.1% bovine serum albumin and 0.5 mL of 20% trichloroacetic acid. The insoluble material obtained by centrifugation at 10,000 rpm for 5 min (Eppendorf, Germany) was analyzed in a gamma counter (Berthold, Germany).

### 2.11 Biodistribution and pharmacokinetics of C-PC

Lewis rats were used to evaluate the biodistribution and pharmacokinetics of C-PC administered by four different routes: intraperitoneal (ip), intravenous (i.v.), intranasal (i.n), and oral. For the i.v. and i.n. routes, a unique dose of 1mg/kg was used. The samples were taken at 1, 4, 8, 12, and 24 h post-administration in the five rats of this subgroup. For the i.n. scheme, the samples were also collected at 1, 10, and 30 min after receiving the compound, in addition to long-time sampling at 1, 2, 4, and 24 h. In all experiments, tissue distribution was determined at 24 h by euthanasia under narcosis overdose. Percent of accumulated dose per sample was calculated with respect to 1 mL of standard dilution of administered dose measured in the same condition in a well-type scintillation counter calibrated for the energy of ^125^I. Results are expressed as percent of uptake relative to total radioactivity dose (%D) or percent of uptake per mass of tissue (%D/g).

Pharmacokinetic analysis was carried out following the non-compartmental approach using Pkanalix (Monolix Suite 2021R2, Lixoft, France).

### 2.12 Cell culture and experimental groups

Human SH-SY5Y cells were maintained in culture DMEM/F12 medium supplemented with fetal bovine serum, penicillin, streptomycin, and L-glutamine. The study was divided into three groups, each one containing 4 x 10^6^ SH-SY5Y cells: 1) non-treated cells (used as a control), 2) PCB plus Glutamate, and 3) Glutamate. After 24 h of pre-stimulation with 0.1 µM PCB, the medium was replaced with freshly prepared 0.01 µM PCB plus 60 µM Glutamate (group 2) or 60 µM Glutamate alone (group 3) for another day. Afterward, the medium was removed; cells were washed using cold PBS and processed to conduct proteomic expression analysis. Three biological replicates were used per group.

### 2.13 Differential protein expression

Proteins were extracted in 1.5% SDS/50 mM DTT with boiling for 10 minutes. Samples were filtered by FASP according to Wisniewski (53) after the reaction of cysteines with iodoacetamide. Overnight Lysyl endopeptidase and 6 h Trypsin digestions were performed at 37 °C. Samples (1 μg) were analyzed in a Thermo Exploris 480 mass spectrometer via LC-ESI-MS/MS. A nanoLC Ultima 3000 coupled through a Pepmap column (75 μm x 150 mm) to the MS was used. Gradients of 80% acetonitrile in 0.1% formic acid were performed in 120 min at 300 nL/min flow rate. The mass spectrometer was operated in data-dependent analysis (DDA) mode with dynamic exclusion of 30 s and full-scan MS spectra (m/z 350–1650) with a resolution of 120,000 (m/z 200), followed by fragmentation of the most intense ions within 1 s cycle time with high energy collisional dissociation (HCD), normalized collision energy (NCE) of 30.0, and resolution of 15,000 (m/z 200) in MS/MS scans.

Identification of peptides and proteins was based on the match-between-runs procedure using MaxQuant software (v1.6.14.0) (54), considering oxidation (M), deamidation (NQ), and N-terminal acetylation as variable modifications. Alignment of chromatographic runs was allowed with a 20-min alignment window and a time matching of 5 min between runs. Filtering and quantification were performed in the Perseus computational platform (v1.614.0) (54). Student’s t-test was employed to identify statistically significant changes (p-values lower than 0.05) in protein levels, after filtering for two valid values in each group.

### 2.14 Bioinformatics analysis

Differentially modulated proteins associated with inflammation, pain, arthritis, neurodegenerative diseases, and glutamatergic transmission were identified by a literature search in the Pubmed database (https://pubmed.ncbi.nlm.nih.gov/). To retrieve the information contained in Pubmed, the text mining tools Chilibot (chip literature robot) (http://www.chilibot.net/) and GeneCUP (https://genecup.org/) were used (55, 56). The data mining study was complemented with the information retrieved from the Diseases 2.0 database (https://diseases.jensenlab.org). Such a database provides confidence scores to disease-gene associations annotated using text mining and data integration tools (57). Diseases related to differentially modulated proteins were also retrieved from DisGeNET (https://www.disgenet.org/) and GAD_Disease databases by using the DAVID functional annotation tool (https://david.ncifcrf.gov/) (58, 59). Interactions among differentially modulated proteins were retrieved using the STRING database (http://string-db.org/) (60). In such analysis, all STRING interaction sources were selected and the confidence score was fixed at 0.4. The biological network of functional associations was visualized using Cytoscape software (v.3.5) (61).

### 2.15 Statistical analysis

The statistical analysis was carried out with the GraphPad Prism software version 9.5.1 (GraphPad Software Inc., CA, USA). All data was expressed as the mean ± standard error of the mean (S.E.M.). Data from the control and the AIA + vehicle groups obtained from different experiments were pooled for the statistical analysis of the hypernociception, the neutrophil quantification, and the MPO activity. The normality of the data was assessed, and when appropriate, it was analyzed by one-way ANOVA and Tukey’s multiple comparisons test (parametric). Non-Gaussian measurements were analyzed by Kruskal-Wallis and Dunn’s multiple comparisons tests (non-parametric). Differences between groups were considered statistically significant at p<0.05 (**Supplementary Table S2**).

## 3 Results

### 3.1 C-PC ameliorates AIA-induced injury

We started our study with the evaluation of C-PC in the AIA mice model, with the assumption that this biliprotein acts as a prodrug during its *in vivo* administration by releasing the pharmacologically active compound PCB into the body. The tetrapyrrole PCB is linked to the specific cysteine residues in C-PC through thioether linkages. Its unbinding could be by means of enzymatic activities as proteases in the form of short peptides or inclusive by the direct rupture of the thioether bond. Another form implies the acidic pH in the stomach, in which this chemical bond is unstable. Nevertheless, the PCB could be linked to another plasmatic protein (i.e., albumin) to facilitate its transport to target cells.

**Figure 1:**
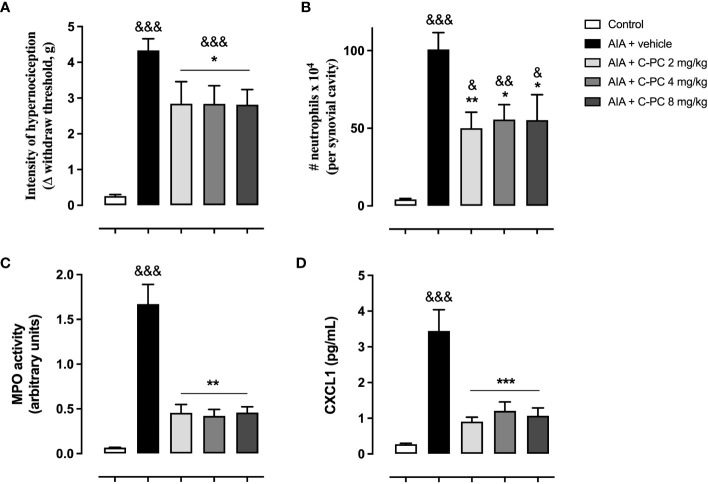
C-Phycocyanin reduces the hypernociception **(A)**, neutrophil infiltration **(B)**, MPO activity **(C)**, and CXCL1 levels **(D)** in AIA mice. Data are mean ± SEM of 5-11 mice/group. *p<0.05, **p<0.01, ***p<0.001, compared with vehicle-treated arthritic mice; ^&^p<0.05, ^&&^p<0.01, ^&&&^p <0.001, compared with control (ANOVA + Tukey’s tests).

As observed in **Figure 1A**, the AIA mice that received the vehicle treatment presented a significantly increased nociception in comparison with the control group. The prophylactic administration of either of the three doses of C-PC (2, 4, or 8 mg/kg) was able to significantly reduce the hypernociception in AIA mice one day following the antigen challenge, in comparison with the AIA + vehicle (**Figure 1A**). AIA also produced a notable increase of neutrophil infiltration, as well as MPO activity in the periarticular tissue of vehicle-treated mice (**Figures 1B, C**). The treatment with C-PC at the three doses evaluated significantly curtailed the entrance and accumulation of neutrophils in the affected synovial cavity (**Figure 1B**) and the MPO activity (**Figure 1C**). In addition, C-PC significantly diminished the CXCL1 chemokine concentrations at all doses assessed with respect to the diseased animals that received the vehicle. (**Figure 1D**). It is noteworthy that a dose-response effect for the range of doses of C-PC evaluated was not observed.

### 3.2 Biodistribution and pharmacokinetics of C-PC

**Figure 2:**
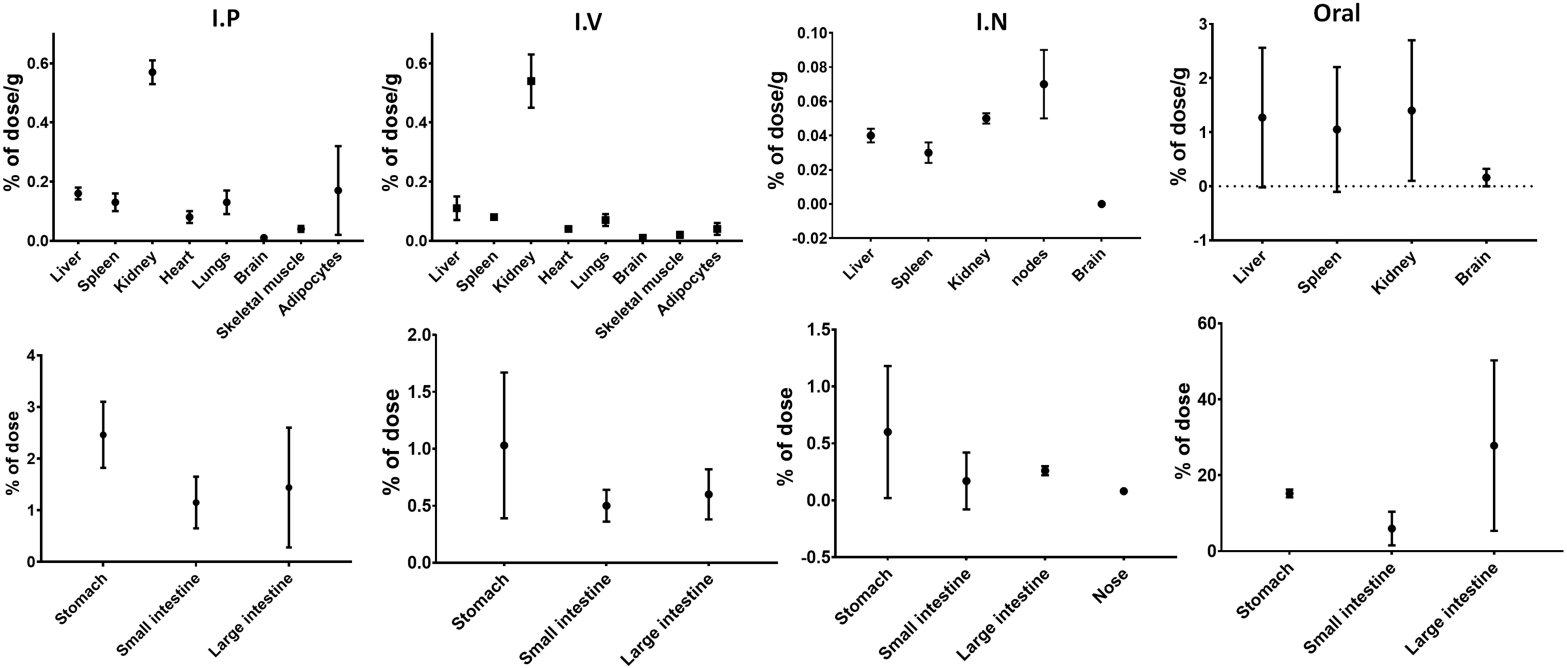
Distribution of C-PC after intraperitoneal (n=4), intravenous, nasal (n=2), and oral (n=2) administration in Lewis rats. Values expressed as %D/g of tissue or %D relative to total dose and standard deviation.

A radioactive assessment was conducted to determine the C-PC’s biodistribution in various tissues. In the i.p., i.v., i.n., and oral routes, radioactivity was detected at levels representing less than 2% of the total administered dose. Conversely, as expected upon oral administration, the accumulation of C-PC was notably higher within the digestive tract, particularly in the large intestine (**Figure 2**). The pharmacokinetic analysis revealed that i.v. administration led to an exponential decline in plasma C-PC levels. This decay was characterized by an average clearance of 8.9 mL/h and a distribution volume at the stationary phase of 295 mL, which closely matched the body mass of the experimental subjects. Elimination was nearly complete 24 h after i.v. administration, as indicated by an area under the curve of 22.7 h _*_ μg/mL. Oral and i.n. administrations demonstrated similar trends in the plasmatic C-PC levels, with the maximum concentration reached at 1 and 4 h, respectively. However, the maximum concentration was much higher in the case of i.n. administration, reaching the value of 12.0 μg/mL, compared to the value of 6.8 μg/mL achieved by the oral route at these time points. Indeed, when comparing the areas under the curve, a clear increase was evident for the i.n. route with 226 h _*_ μg/mL, whereas for the oral route, it was 77 h _*_ μg/mL (**Supplementary Figure S1**).

### 3.3 Dose-response effects of PCB against hypernociception and neutrophil infiltration in mice with AIA

**Figure 3:**
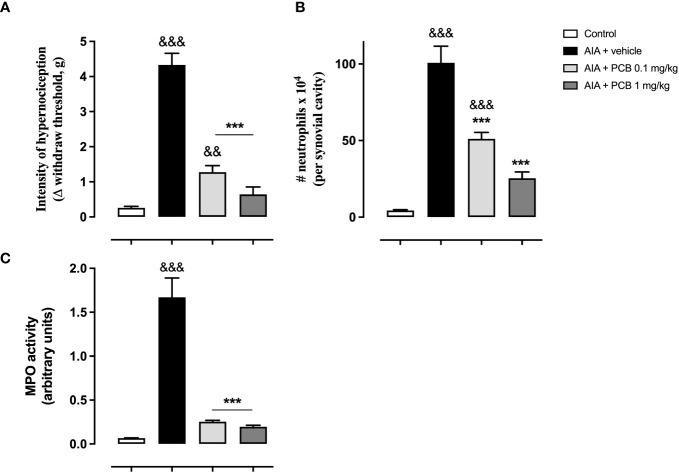
Phycocyanobilin ameliorates the hypernociception **(A)**, the neutrophil accumulation **(B)**, and the MPO activity **(C)** in mice with acute AIA. Data are mean ± SEM of 5-11 mice/group. ***p<0.001, compared with vehicle-treated arthritic mice; ^&&^p<0.01, ^&&&^p <0.001, compared with control (ANOVA + Tukey’s tests).

The prophylactic PCB treatment was effective in ameliorating the arthritis-induced hypernociception in mice following one day of antigen challenge, with respect to the AIA animals receiving the vehicle (**Figure 3A**). The quantification of the leucocyte infiltration into the inflamed knee revealed that the pretreatment with PCB significantly lessened the neutrophil agglomeration in the synovial space, showing a response associated with the used doses, when statistically contrasting with the vehicle AIA mice (**Figure 3B**). Similarly, PCB produced a decline in the MPO activity in relation to the AIA mice that were injected with vehicle solution. (**Figure 3C**). Importantly, this effect of PCB followed a dose-response behavior.

### 3.4 PCB effects on cytokine production and T cell markers expression in mice with AIA

**Figure 4:**
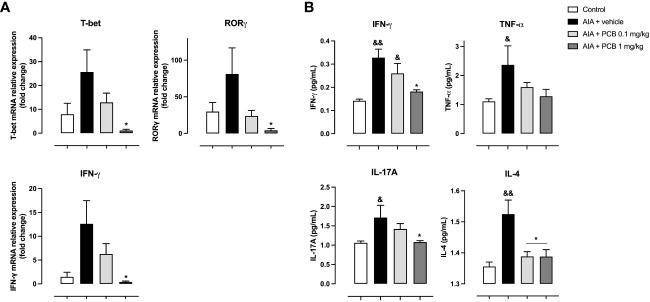
Effect of Phycocyanobilin on **(A)** mRNA levels by qPCR in popliteal lymph nodes, and **(B)** protein levels by Cytometric Bead Array (CBA) in the supernatant from periarticular tissues homogenates. Data are mean ± SEM of 5-6 mice/group. *p<0.05, compared with vehicle-treated arthritic mice; ^&^p<0.05, ^&&^p<0.01, compared with control. ANOVA + Tukey tests for IFN-γ (mRNA and protein), T-bet, RORγ, and IL-4; Kruskal-Wallis + Dunn tests for TNF-α and IL-17A.

On the other hand, a significant downregulation of PCB at 1 mg/kg on the transcriptional factors T-bet (Th1), RORγ (Th17), and the proinflammatory cytokine IFNγ was observed compared with the AIA + vehicle group, as determined by qPCR analysis of popliteal lymph nodes (**Figure 4A**). Likewise, PCB treatment dose-dependently restricted the expression of cytokines mediating a proinflammatory Th1 phenotype in periarticular tissue homogenate as assessed by CBA: IFN-γ with a significant difference and a clear trend towards a reduction for TNF-α when the AIA + PCB 1 mg/kg was compared with the AIA + vehicle (**Figure 4B**). Furthermore, a significant drop was also achieved with the treatment of 1 mg/kg PCB, the uppermost dose used, for the cytokine IL-17A (**Figure 4B**). Interestingly, treatment with both PCB amounts tested (0.1 and 1 mg/kg) produced a significant reduction of the cytokine IL-4, characteristic of a Th2 response, in AIA mice compared with diseased animals receiving the vehicle (**Figure 4B**).

### 3.5 PCB reduces the tissue damage in the lesioned joint of mice with AIA

**Figure 5:**
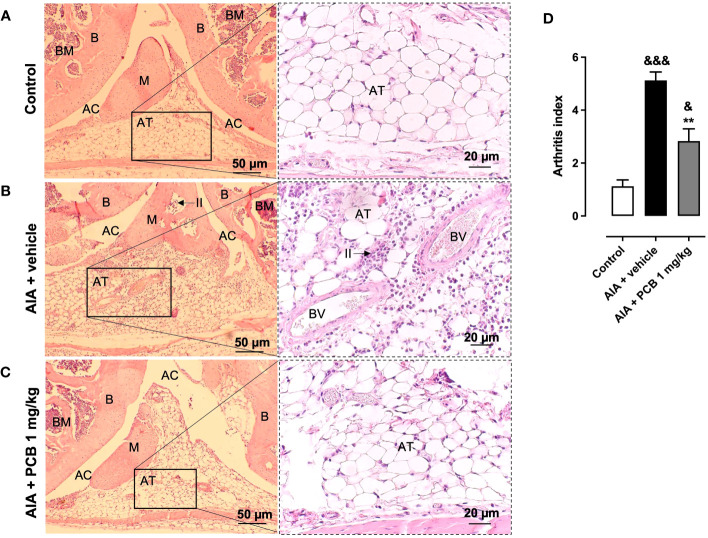
Histological assessment of PCB on the knee joint in AIA mice. Photographs are representative of **(A)** Control, **(B)** AIA + vehicle, and **(C)** AIA + 1 mg/kg PCB. **(D)** Quantitative evaluation of PCB effects on the arthritic index. The right panels are magnified 2.5 times the respective region in the left panel for **(A-C)**. Histopathological confirmation of the AIA-caused joint inflammation is evident on panels **(B)**, such as the occurrence of inflammatory infiltrate (II), indicated by arrows, in the adipose tissue (AT) and inside the meniscus (M). It is also observed in panels **(B)** an increase in blood vessels (BV) with their red blood cells inside the AT and the modification of tissue morphology when examining in relation to the other panels **(A, C)**. AT, Adipose Tissue; AC, Articular Cavity; B, Bone; BM, Bone Marrow; M, meniscus; and II, Inflammatory Infiltrate. Data are mean ± SEM of 4-6 mice/group. **p<0.01, compared with vehicle-treated arthritic mice; ^&^p<0.05, ^&&&^p <0.001, compared with control (ANOVA + Tukey’s tests).

Based on this encouraging evidence, we then decided to perform a histological assessment of the affected knees to confirm the protective activity of PCB on the tissue structure. The healthy control is shown in panel 5A (**Figure 5A**). On the contrary, those animals affected by the disease and receiving the vehicle treatment showed a rise of the arthritis index, with a distinctive synovial accumulation of polymorphonuclear cells (**Figure 5B**). This feature was counteracted by 1 mg/kg PCB, which dramatically reduced the leucocyte exudate in the joint space (**Figure 5C**) Accordingly, the arthritis index showed a significant reduction in diseased animals treated with PCB at 1 mg/kg compared with AIA + vehicle group (**Figure 5D**).

### 3.6 PCB regulates the proteome profile in glutamate-exposed SH-SY5Y neuronal cells

**Figure 6:**
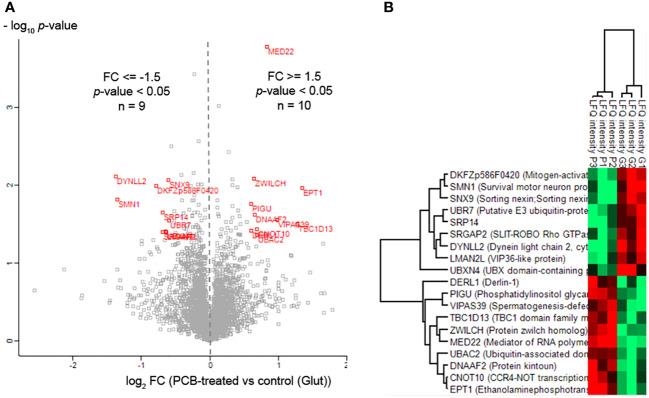
Proteomic profile modulated by PCB in SH-SY5Y cells. **(A)** Volcano plot of quantified proteins from SH-SY5Y cells after PCB treatment. Red points indicate those proteins that met the statistical significance cutoff (|FC| ≥ 1.5; *p*-value<0.05). **(B)** Hierarchical clustering of proteins differentially modulated by PCB treatment compared to cells subjected to Glutamate damage (three replicates per condition). Red and green colors mean upregulated and downregulated, respectively.

To identify the array of proteins under the regulation of PCB, we conducted quantitative proteomic analysis on SH-SY5Y neuronal cells. These cells were exposed to the excitatory neurotransmitter glutamate and subsequently treated with or without PCB over a 24-hour period. Out of a total of 4,511 identified proteins, 19 proteins displayed differential modulation in cells treated with PCB (**Table 1**, **Figure 6**, and **Supplementary Table S3**). An additional comparative study was performed to quantify the proteins modulated by either the PCB treatment or the glutamate injury independently by using non-treated SH-SY5Y cells as the control condition (**Supplementary Table S4** and **Supplementary Figure S2**).

**Table 1:** Differentially modulated proteins SH-SY5Y cells treated with PCB.

UniProt _ACC *^a^*	Description	Gene Symbol *^b^*	FC *^c^*	*p*-value *^c^*
PCB up-regulated proteins				
Q9C0D9	Ethanolaminephosphotransferase 1	EPT1	1.35	1.96
Q9NVG8	TBC1 domain family member 13	TBC1D13	1.27	1.49
Q9H9C1	Spermatogenesis-defective protein 39 homolog	VIPAS39	0.98	1.55
Q15528	Mediator of RNA polymerase II transcription subunit 22	MED22	0.83	3.77
Q8NBM4	Ubiquitin-associated domain-containing protein 2	UBAC2	0.69	1.36
Q9H9A5	CCR4-NOT transcription complex subunit 10	CNOT10	0.69	1.43
Q9NVR5	Protein kintoun	DNAAF2	0.65	1.61
Q9H900	Protein zwilch homolog	ZWILCH	0.64	2.08
Q9BUN8	Derlin-1	DERL1	0.60	1.42
Q9H490	Phosphatidylinositol glycan anchor biosynthesis class U protein	PIGU	0.60	1.76
PCB down-regulated proteins				
Q8N806	Putative E3 ubiquitin-protein ligase UBR7	UBR7	-0.59	1.54
Q9Y5X1	Sorting nexin;Sorting nexin-9	SNX9	-0.60	2.06
Q9H0V9	VIP36-like protein	LMAN2L	-0.64	1.39
Q92575	UBX domain-containing protein 4	UBXN4	-0.64	1.41
H0YLW0	Signal recognition particle 14	SRP14	-0.69	1.64
O75044	SLIT-ROBO Rho GTPase-activating protein 2	SRGAP2	-0.69	1.40
O43318	Mitogen-activated protein kinase kinase kinase 7	MAP3K7	-0.78	1.99
Q16637	Survival motor neuron protein	SMN1	-1.34	1.81
Q96FJ2	Dynein light chain 2, cytoplasmic	DYNLL2	-1.37	2.11

^a^ Accession numbers in the UniProtKB database.

^b^ Recommended gene name (official gene symbol) as provided by UniProtKB.

^c^ Fold change (FC) and p-values of differentially modulated proteins according to quantiﬁcation performed in Perseus computational platform (v1.614.0) [ (–) downregulated].

**Figure 7:**
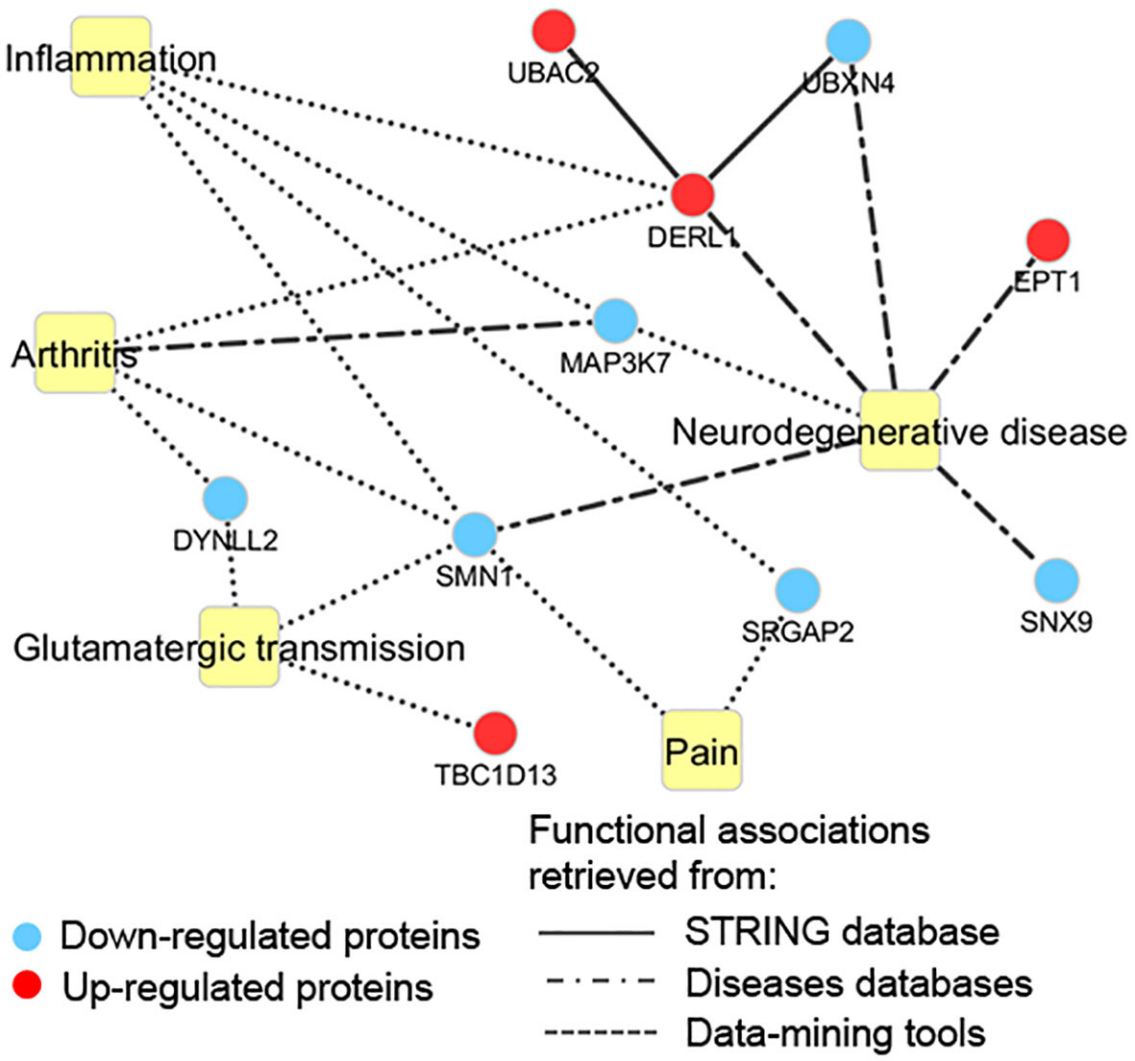
Functional terms associated with the proteomic profile modulated by PCB. Edges types are represented according to the data source.

For a better understanding of putative cellular processes affected by PCB, the 19 differentially modulated proteins were classified using the information from disease databases and text mining tools (**Supplementary Table S4**). As shown in **Figure 7**, besides proteins related to neurodegenerative diseases, PCB treatment downregulates proteins involved in arthritis and pain. Furthermore, proteins that play a role in glutamatergic transmission and inflammation were also modulated in response to PCB treatment.

## 4 Discussion

The first-ever published observations regarding the beneficial actions of *Spirulina* extracts against arthritis were published in 2002 by a Cuban group in a model of this disease in mice induced by zymosan (62). This group attributed the observed effect to C-PC, which constitutes around 15% of *Spirulina* dry biomass (63). Our group has studied the actions of C-PC in animal models of multiple sclerosis, such as the Experimental Autoimmune Encephalomyelitis, in which we have reported positive outcomes of this biliprotein, in a manner dependent on the dose, in the range of 2, 4, and 8 mg/kg, when administered intraperitoneally (64). Using the same experimental setting, we tested three PCB amounts (0.1, 0.5, and 1 mg/kg), and a dose-response positive activity was also obtained (65). Thus, previous observations have confirmed that PCB is the main responsible agent for the biological activities of C-PC (66), as demonstrated in different experimental scenarios such as ischemic stroke (66) and acute kidney damage (67). Following this reasoning, we first intended to evaluate if the administration of C-PC could alleviate AIA in mice. Then, we questioned whether the application of PCB may counteract the AIA-induced injury by assessing different outcomes: functional (hypernociception), immunological (neutrophils and cytokines), biochemical (MPO activity), gene expression, and histopathological. The doses of PCB used in the present study were calculated in function of the C-PC composition. As mentioned above, this biliprotein contains three PCBs attached. Although the lower PCB dose evaluated (0.1 mg/kg) is equivalent to 2 mg/kg C-PC, we highlight the fact that the higher PCB dose used in this study (1 mg/kg) is equivalent to a 2.5-fold increase of 8 mg/kg C-PC (higher evaluated dose). Since a dose-response effect was not observed with these C-PC administered amounts, we increased the PCB dose to better detect the dose dependence of its pharmacological activity against AIA.

Our observations confirmed a significant C-PC-promoted reduction of pain and joint inflammation in AIA mice. Reports of anti-arthritic properties of this compound have been demonstrated with other arthritis models. These anti-arthritic properties of the compound are attributed to its selective inhibitory property on COX-2, its efficacy in eliminating free radicals, and inhibiting lipid peroxidation (68). C-PC can prevent osteoarthritis by attenuating the oxidative stress in chondrocytes induced by H_2_O_2_ (69). Interestingly, a similar property has also been reported by our group for the PCB treatment inhibiting the H_2_O_2_ damage in PC12 cells (70). C-PC has been shown to act on fundamental processes such as inflammation, chondral degeneration, and oxidative stress in canine *in vitro* osteoarthritis (71). Our study evidenced that C-PC significantly reduced the alpha chemokine CXCL1 in the AIA model. CXCL1 has been shown to have neutrophil chemoattractant activity in inflamed tissue in arthritis following activation of the CXCR2 receptor (72). Notably, antagonism of this CXCR2 receptor has resulted in an inhibition of neutrophil migration strongly associated with reduced injury in rats experiencing AIA (73). In patients with RA, the rise of CXCL1 expression correlated with the accumulation of neutrophils (74). This evidence suggests that the inhibition of the CXCL1 expression could mediate the protective actions of C-PC in AIA mice, via the decreased infiltration of neutrophils to the injured joint tissue.

The biodistribution of C-PC following i.v., i.p., and i.n. administration exhibited notably low levels across all examined tissues. However, in the case of oral administration, significant levels of the radioactive-labeled product were detected along the entire digestive tract. Notwithstanding, at least 50% of the product appears to be absorbed and redirected by blood flow. This result is consistent with the way that the molecule goes to its target sites. An important point to consider is that radiolabeling occurs mainly at the residues His and Tyr of the protein by nucleophilic aromatic substitution (75). This reaction is not possible in PCB due to the absence of aromatic carbon-hydrogens as part of its structure. However, PCB is released from C-PC after proteolysis (76), and it could reach the bloodstream and be transported throughout the body in solution given its stability (77) or attached to serum albumin (78), in a chemical association that appears to be mutually protective against oxidative damage, keeping the antioxidant activity and the chemical structure of this tetrapyrrole.

Furthermore, the pharmacokinetic results are in line with the significant chemical and biological disparities between the digestive tract and the nasal route. As mentioned above, oral administration of C-PC is followed by the degradation of its polypeptide moieties, resulting in the liberation of PCB. This tetrapyrrole has been identified as the active molecule accountable for the observed pharmacological effects of C-PC (67). Consequently, it becomes plausible that the radioactivity detected in plasma after oral administration comprises short peptides covalently linked to PCB. Supporting this observation are the clearance values of 5.6 mL/h following the i.n. administration and 21.0 mL/h for the oral route. Such elevated clearance values are indicative of compounds with lower molecular weights. Additionally, the potential shielding effect of the polypeptide moieties on the prosthetic group could explain the prolonged retention in plasma of the initially administered protein through the i.n. route. Taken together, the biodistribution and pharmacokinetics behavior of C-PC across different routes of administration contribute to understanding how this compound, and its derivative PCB, interacts with different tissues and how the body processes it. This essential data can also guide future dosing strategies and the design of drug delivery systems to ensure that the drug reaches its intended target. In this line of thinking, we can envision the application of nanotechnology for improving the controlled delivery of PCB toward targeted tissues. The nanotechnology-enabled PCB delivery systems, such as liposomes or nanoparticles, can enhance the bioavailability and stability of this tetrapyrrole, leading to prolonged therapeutic effects in RA (79).

RA is a pathology associated with impairment in the redox and immunological balances, and neutrophils are key mediators in both processes (80). In this study, we observed that both molecules, C-PC and PCB, limit the severity of AIA due to, at least in part, inhibiting the migration of neutrophils to the affected synovial cavity. In this context, neutrophils may directly damage knee structures, such as the bone and the cartilage, through the production of proteases and reactive oxygen species (ROS). Additionally, neutrophils could also dictate some inflammation processes either by antigen presentation or by the release of soluble factors, including prostaglandins, chemokines, cytokines, and leukotrienes (81). Dysregulated activation of neutrophils and their derived ROS production are implicated in tissue damage and destruction in RA (82).

The distinctive harm caused by RA to the cartilage and bone of the knee is associated with synovial exudate of immune cells and a disproportionate increase of proinflammatory factors such as TNF-α, IFN-γ, and IL-17A (83, 84). Our results demonstrated that a PCB-mediated effect was detected by qPCR on the T-bet (Th1 phenotype) and RORγ (Th17 phenotype) transcriptional factors expression, as well as IFN-γ cytokine in popliteal lymph nodes. At the protein level, PCB also reduced the concentration of the proinflammatory cytokines TNF-α, IFN-γ, and IL-17A in periarticular tissue.

IFN-γ and IL-17A are versatile cytokines widely involved in inflammatory diseases, mediating both immune activation and tolerance. In our study, a protective role of PCB in AIA mice was demonstrated, which could be associated with a reduction of both cytokines, critical components of the inflammatory damage induced by AIA (85).

These cytokines were measured in periarticular tissue homogenate, suggesting that a diverse set of immune cells, other than neutrophils, may also be locally involved in the AIA-induced acute synovial inflammation. Our observations point to an involvement of IL-17A-producing T cells, known as Th17 cells, in this pathological scenario. Accumulated evidence has shown the pivotal role of Th17 cells in diverse pathological conditions encompassing the immune system. These specialized cells have demonstrated their involvement in various autoimmune disease models (86). Notably, they have been linked to the progression of experimental autoimmune encephalomyelitis (EAE) (87), and collagen-induced arthritis (88). Furthermore, Th17 cells facilitate the recruitment of neutrophils in airways and in collagen-induced arthritis (89, 90), contributing to the perpetuation of the inflammatory milieu in arthritis. Moreover, Th17 plays a significant role in osteoclastogenesis, as it efficiently upregulates the expression of RANKL on osteoclast precursors (91, 92). This molecular modulation sets the stage for the induction of osteoclast formation and bone resorption, contributing to the pathogenesis of arthritis.

Th17 cells *in vitro* differentiation from naïve CD4^+^ T subset is dependent on the cytokines TGF-β, IL-6, and IL-23 via induction of the transcription factor RORγ (93). We have previously demonstrated that PCB inhibits the proliferation of antigen-activated encephalitogenic CD4^+^ T cells in rats and in 2D2 mice (56). This supports the results of the current study, in which we observed a downregulation effect of PCB on the Th17 master transcriptional factor RORγ in popliteal lymph nodes. Thus, PCB may inhibit the proliferation of naïve activated CD4^+^ T precursor cells leading to a reduced number of differentiated Th17 in the periphery, which consequently will provoke their limited migration into the affected joint. In addition, it has been demonstrated an inductor activity of C-PC/PCB on the differentiation of regulatory T cells (Treg) (94). Treg inhibits the activity and differentiation of Th17 cells and favors the amelioration of experimental inflammatory arthritis (95, 96).

On the other hand, over the past few years, intriguing findings have advanced the insight of neutrophils from mere short-lived first responders of the innate immune system, towards active participants in adaptive immunity, particularly in chronic inflammatory disorders such as RA (97). In this newfound role, neutrophils serve as accomplices, not just bystanders of cells from the adaptive immune response such as T cells and DCs. The decisive role of neutrophils in guiding the trajectory of T-cell development, orchestrated by DCs, towards Th17 cells, relies on potent factors present within their granule content, which include neutrophil elastase (98) and lactoferrin (99). Our data suggest that by inhibiting the neutrophil accumulation and activity in the inflamed joint, C-PC/PCB may indirectly curtail the Th17 cell development driven by the interaction between neutrophils and DCs.

Furthermore, a direct effect of PCB on the function of human monocyte-derived DCs has been reported. Pre-treatment of human DCs *in vitro* with PCB before stimulation with lipopolysaccharide led to a significant reduction in the supernatant levels of cytokines IL-12p70 and IL-23, accompanied by a reduced expression of costimulatory molecules CD83 and CD40 (100). When these lipopolysaccharide-stimulated DCs were treated with PCB and cocultured with allogeneic CD4^+^ T cells, Basdeo et al. (2016) observed a significant decrease of more than 50% in the production of IFN-γ, which is characteristic of Th1 cell polarization, compared to the untreated control (89). This evidence suggests that PCB halts the maturation of DCs, leading to a drop in their production of polarizing cytokines, ultimately downregulating the adaptive inflammatory response. Thus, PCB may also prevent the development of synovial inflammation by directly inhibiting the professional antigen-presenting functions of DCs recruited into the affected synovium (101), along with the consequent reactivation of CD4^+^ T cells (102).

It is noteworthy to mention our results indicating the decreasing effect of both PCB doses on the cytokine IL-4, a cytokine that is known for its anti-inflammatory properties, is produced by Th2 and Tc2 cells, but the action of IL-4 on other types of T cells, such as CD8+, Tregs, and Th9 T cells, has been also described. Furthermore, a versatile effect of IL-4 is known on B cells (103). The regulatory function of IL-4 on inflammation depending on the context of RA is known (104). Though this cytokine is frequently reported to mediate anti-inflammatory events, its proinflammatory actions have also been described as probably controlled by Th cells. This biphasic IL-4 activity could, therefore, restrain immunity when cellular components are involved, while fostering the humoral component of the immune response, in a context-specific manner (105). Interestingly, the IFN-γ/IL-4 ratio (106) could better express the Th1/Th2 balance that explains the effect of PCB on AIA.

In addition to the biological actions of PCB here observed, this organic molecule presents other advantages in relation to C-PC when considering its pharmaceutical development (1): it can be obtained either by chemical synthesis (107, 108) or by genetic engineering of its synthesizing enzymes in *E. coli* (109) (2), chemical products such as PCB avoid exhaustive bioequivalence studies because a well physicochemical characterization is enough to introduce it as therapeutic agents (3), PCB could be attached to protein drugs in combined therapies (4), PCB is more stable than C-PC under proteases attack, and (5) due to the small size of PCB (relative to C-PC), it may overpass many body compartments prohibited to large macromolecules, such as the blood-brain barrier. Because of all of the above, we decided to carry out our experiment with this tetrapyrrole to evaluate its effects as a possible treatment for RA.

In this study, we performed a proteomic profiling of glutamate-exposed SH-SY5Y neuronal cells as an *in vitro* model to evaluate the effects of PCB on one of the main excitatory signaling in nociception and neurodegeneration. The modulation of proteins related to biological processes such as inflammation, glutamatergic transmission, and pain were evaluated. Those associated with pathologies such as arthritis and neurodegenerative diseases were also considered. After the analysis, we found that only 19 proteins were differentially modulated in SH-SY5Y cells treated with both PCB and glutamate. It is worth noting that the concentration of glutamate used (60 μM) was relatively high (110, 111), which may be a limitation of the present study. However, the evidence obtained may have opened new insights regarding the PCB effects on neuronal excitatory states.

Through a protein network analysis, we identified that the proteins influenced by PCB treatment were interconnected with diseases and implicated in biological processes. One notable finding was the PCB downregulation of Mitogen-activated protein kinase 7 (MAP3K7) or a ubiquitin-dependent kinase of MKK and IKK (TAK1) (112). MAP3K7 is a serine/threonine kinase that integrates many biochemical signals. This protein is involved in numerous cellular processes such as proliferation, differentiation, transcriptional regulation, and development. As a part of the mechanism of this kinase, after activation by extracellular stimuli, it translocates to the nucleus and regulates gene expression by phosphorylating various transcription factors (113, 114). MAP3K7 has also been strongly implicated in many of the processes underlying the pathology of rheumatoid arthritis (115, 116) and neurodegenerative diseases (117) in which inflammation (118, 119) is implicated. The current findings suggest that MAP3K7 downregulation may also contribute to the PCB effects on the reduction of neuroinflammation-induced microglial dysfunction as was identified in our previous study. Furthermore, MAP3K7 plays a crucial role in pain signaling from various causes, including inflammation. Notably, the utilization of the MAP3K7 inhibitor takinib demonstrated its capacity to mitigate inflammatory, neuropathic, and primary pain, in animal models established through intraplantar administration of complete Freund’s adjuvant, chronic constriction injury, and systemic introduction of catechol-O-methyltransferase, respectively (120). MAP3K7 inhibition was also able to diminish the expression of pain-associated mediators in synovial cells isolated from arthritis patients, suggesting its important role in managing peripheral sensitization in arthritis nociception (121). Therefore, by downregulating MAP3K7 expression, PCB may act on the amelioration of arthritis pain.

Neutrophils in the synovial fluid of patients with arthritis exhibit a reduced ability to suppress activated T cells, possibly related to changes in proteins involved in cell-cell contact and inflammation (122). Among the proteins involved in the interaction between neutrophils and T cells is Dynein light chain 2 (DYNLL2) (123), which regulates the dynein 1 function and subsequently the cargo and movement of vesicles and organelles through microtubules (124). This protein has also been documented to play a central role in the interaction of the NMDA receptor-associated scaffold complex and the enhancement of the synaptic NMDA receptor activity (125). In our study, we identified a downregulation of the DYNLL2 protein in SH-SY5Y cells treated with PCB. One possible explanation could be associated with key elements in the mechanisms that underlie the protective effects of PCB, such as the reduction of oxidative stress through the inhibition of NADPH oxidase (42) and the amelioration of inflammation.

Another protein that displayed downregulation was the Survival Motor Neuron Protein 1 (SMN1), a constituent of a complex catalyzing the assembly of small nuclear ribonucleoproteins (snRNPs). The expression of this protein was associated with levels of inflammatory cytokines (IL-1β and TNF-α) in osteoarthritis (126). Thus, the limited expression of SMN1 induced by PCB may contribute to keeping the levels of these proinflammatory cytokines during arthritis under control, as a previously observed effect of PCB on these cytokines in a model of experimental autoimmune encephalomyelitis (65). On the other hand, PCB also induced a downmodulation of SLIT-ROBO Rho GTPase-activating protein 2 (SRGAP2) in SH-SY5Y cells treated with glutamate. This protein participates in neuronal morphogenesis and is also linked with neuronal migration during cerebral cortex development (127, 128). In this context, it has been reported that the elimination of endogenous expression of SRGAP2 promotes neurite growth in differentiated cells (129) and contributes to the restriction of osteoclastogenesis during arthritis-related inflammation (130).

Another modulated process was identified as mediated by Derlin-1 (DERL1), a protein that serves as a functional component of endoplasmic reticulum-associated degradation of misfolded proteins (131). Its disruption hampers neurite outgrowth and nervous system development, ultimately leading to brain atrophy (132). In our study, we observed an upregulation of the DERL1 in glutamate-exposed SH-SY5Y cells treated with PCB, which may impact functions related to the endoplasmic reticulum. One study reported that DERL1 colocalized with neurofibrillary tangles in the brain of patients with Alzheimer’s disease and could play an important role in endoplasmic reticulum-associated neurodegeneration (133). Another protein that has been shown to interact with DERL1 is the mutated superoxide dismutase 1 (SOD1), which accumulates and misfolds in motor neurons in amyotrophic lateral sclerosis (134), another neurodegenerative disease, and the interaction with DERL1 is involved in the disease pathophysiology (135). Another DERL1 interacting protein is the NADPH oxidase subunit p22phox, and a previous report has shown that this interaction regulates the partner degradation of p22phox, with potential implications in the reactive oxygen species-producing capacity of this enzyme (136). This accumulated evidence suggests that PCB could potentiate the adequate functioning of endoplasmic reticulum quality control by upregulating DERL1, and thus preventing the associated cellular dysfunction (132). In this sense, it has been suggested that inhibitors of endoplasmic reticulum stress could mitigate chondrocyte damage and reduce arthritis degeneration (137).

Another interesting result of our study was the upregulation of the ethanolamine phosphotransferase 1 (EPT1) protein by PCB in SH-SY5Y cells. This enzyme transfers phosphoethanolamine from cytidine diphosphate-ethanolamine to lipid acceptors to form ethanolamine glycerophospholipids via the ‘Kennedy’ pathway (138). This kinase is part of the enzymes required to synthesize phosphatidylethanolamine within the myelin membrane (139). Studies in a patient with spastic paraplegia revealed hypomyelination and brain atrophy demonstrating its role in the myelination process and in maintaining normal phospholipid homeostasis (140). These studies are consistent with the reported effect of PCB on myelin in an animal model of experimental autoimmune encephalomyelitis (141).

## 5 Conclusion and future perspectives

In summary, the data presented in this study demonstrate that the preemptive administration of C-PC and PCB effectively mitigates mBSA-induced arthritic injuries in mice. This was achieved by limiting hypernociception and reducing neutrophil infiltration and MPO activity, all of which are closely linked to the inflammatory injury occurring in the knee during RA. Moreover, PCB exhibits the ability to modulate the local concentration of proinflammatory cytokines and the inflammatory infiltrate within the affected joint, thereby preserving the structural integrity of the tissue.

The evidence presented here regarding the biodistribution of C-PC opens an intriguing perspective when considering the future administration of its derivative PCB for RA. As follow-up research, exploring the oral route of PCB administration could potentially offer advantages such as improved patient compliance, reduced invasiveness, and enhanced systemic delivery.

The significance of glutamatergic signaling in joint-related pain in RA is well-known. In the context of this study, the integration of bioinformatics tools facilitated the *in vitro* assessment of PCB’s impact on the neuronal proteome when subjected to glutamate excitation. This approach led to the identification of proteins associated with pivotal biological processes including pain, inflammation, and glutamatergic transmission, which hold substantial relevance for pathologies such as RA.

The findings from this investigation point toward a potential application of PCB as an innovative therapeutic approach for RA. Furthermore, the study provides insights into the potential mechanisms of action and therapeutic targets associated with the effects of PCB. Ultimately, these results offer promising avenues for further exploration and development of this novel treatment for RA and related conditions involving inflammatory pain.

## Data availability statement

The original contributions presented in the study are included in the article/**Supplementary Material**. Further inquiries can be directed to the corresponding author.

## Ethics statement

Ethical approval was not required for the studies on humans in accordance with the local legislation and institutional requirements because only commercially available established cell lines were used. The animal study was approved by Federal University of Minas Gerais (UFMG) ethics committee (CEUA UFMG:165/2009). The study was conducted in accordance with the local legislation and institutional requirements.

## Author contributions

JM-P: conceptualization, writing, formal analysis, investigation, and funding acquisition. AR-U: investigation (proteomic analysis) and writing. VB: investigation (proteomic analysis). AL-A: conceptualization, formal analysis and writing. NB: investigation (AIA model). IH-G: investigation (PK/BD studies). NP-F: investigation and funding acquisition. ÉMV: investigation and formal análisis (performance and analysis of flow cytometry). VF-C: investigation (microscopy analysis). EA: investigation (microscopy analysis). GM-D: investigation and project administration. MC-L: investigation (cell culture). DL: investigation (proteomic studies). LG: investigation (proteomic studies) and methodology. JF-M: investigation, formal analysis, and methodology. GG-N: methodology, project administration, and funding acquisition. EP-A: methodology and writing. FA: methodology and writing. MT: methodology, writing, and funding acquisition. GP-R: conceptualization, writing, original draft, project administration, methodology, and funding acquisition. All authors contributed to the article and approved the submitted version.

## Supplementary material

Supplementary Table 3 Proteomic profile regulated by PCB in SH-SY5Y cells.

Supplementary Table 4 Data sources of the functional terms associated with PCB-modulated proteins.

Supplementary Figure 1 Plasma concentration of C-PC after oral (squares), i.n. (circles) and i.v. (triangle) administrations at doses as described in the Material and Method section.

Supplementary Figure 2
**(A)** PCA analysis of protein cell extracts after PCB treatment versus control cells demonstrates how both groups are different; red and blue squares correspond to PCB-treated and non-treated replicates, respectively. **(B)** Volcano plot showing the proteins differentially modulated (p<0.05) after PCB treatment in comparison to control cells. **(C)** Hierarchical clustering of proteins differentially expressed by PCB treatment compared to control cells (three replicates per each condition). Up- and down-regulated genes are in blue and pink colors, respectively.
