# Supplementary material for: The effects of Phycocyanobilin on experimental arthritis involve the reduction in nociception and synovial neutrophil infiltration, inhibition of cytokine production, and modulation of the neuronal proteome
Source: Front Immunol. 2023 Oct 23;14:1227268. doi: 10.3389/fimmu.2023.1227268 (PMC10627171; doi:10.3389/fimmu.2023.1227268)
Supplement: Supplementary file 1 [file Table_1.docx]

| **Gene symbol** | **Full name** | **NCBI Accession No. (GenBank)** | **Forward sequence** | **Reverse sequence** |
| --- | --- | --- | --- | --- |
| Ifng | Interferon gamma | NM_008337 | CAGCAACAGCAAGGCGAAAAAGG | TTTCCGCTTCCTGAGGCTGGAT |
| T-bet | T-bet | NM_019507 | CCACCTGTTGTGGTCCAAGTTC | CCACAAACATCCTGTAATGGCTTG |
| RORgamma | RAR-related orphan receptor gamma | NM_011281 | GTGGAGTTTGCCAAGCGGCTTT | CCTGCACATTCTGACTAGGACG |
| Gapdh* | glyceraldehyde-3-phosphate dehydrogenase | NM_008084 | CATCACTGCCACCCAGAAGACTG | ATGCCAGTGAGCTTCCCGTTCAG |

**Supplementary Table S1.** Primers sequences for qPCR

* Housekeeping gene
