# Supplementary material for: The effects of Phycocyanobilin on experimental arthritis involve the reduction in nociception and synovial neutrophil infiltration, inhibition of cytokine production, and modulation of the neuronal proteome
Source: Front Immunol. 2023 Oct 23;14:1227268. doi: 10.3389/fimmu.2023.1227268 (PMC10627171; doi:10.3389/fimmu.2023.1227268)
Supplement: Supplementary file 2 [file Table_2.docx]

**Supplementary Table S2.** Statistical results obtained for each Figure by using the

GraphPad Prism software version 9.5.1

| **Figure 1.** | **Intensity of hypernociception (Δ withdraw threshold, g)**   \| **One-way ANOVA** \| **SS** \| **DF** \| **MS** \| **F (DFn, DFd)** \| **P value** \| \| --- \| --- \| --- \| --- \| --- \| --- \| \| Treatment (between columns) \| 112.7 \| 4 \| 28.16 \| F (4, 38) = 32.35 \| P<0.0001 \| \| Residual (within columns) \| 33.08 \| 38 \| 0.8706 \|  \|  \| \| Total \| 145.7 \| 42 \|  \|  \|  \|  \| **Tukey’s multiple comparisons test** \| **Mean Diff.** \| **Significant?** \| **Summary** \| \| --- \| --- \| --- \| --- \| \| Control vs. AIA + vehicle \| -4.076 \| Yes \| **** \| \| Control vs. AIA + C-PC 2 mg/kg \| -2.585 \| Yes \| **** \| \| Control vs. AIA + C-PC 4 mg/kg \| -2.580 \| Yes \| **** \| \| Control vs. AIA + C-PC 8 mg/kg \| -2.557 \| Yes \| **** \| \| AIA + vehicle vs. AIA + C-PC 2 mg/kg \| 1.491 \| Yes \| * \| \| AIA + vehicle vs. AIA + C-PC 4 mg/kg \| 1.496 \| Yes \| * \| \| AIA + vehicle vs. AIA + C-PC 8 mg/kg \| 1.519 \| Yes \| * \| \| AIA + C-PC 2 mg/kg vs. AIA + C-PC 4 mg/kg \| 0.005000 \| No \| ns \| \| AIA + C-PC 2 mg/kg vs. AIA + C-PC 8 mg/kg \| 0.02833 \| No \| ns \| \| AIA + C-PC 4 mg/kg vs. AIA + C-PC 8 mg/kg \| 0.02333 \| No \| ns \|   **# neutrophils x 10^4^ (Per synovial cavity)**   \| **One-way ANOVA** \| **SS** \| **DF** \| **MS** \| **F (DFn, DFd)** \| **P value** \| \| --- \| --- \| --- \| --- \| --- \| --- \| \| Treatment (between columns) \| 77127 \| 4 \| 19282 \| F (4, 45) = 22.02 \| P<0.0001 \| \| Residual (within columns) \| 39412 \| 45 \| 875.8 \|  \|  \| \| Total \| 116538 \| 49 \|  \|  \|  \|  \| **Tukey’s multiple comparisons test** \| **Mean Diff.** \| **Significant?** \| **Summary** \| \| --- \| --- \| --- \| --- \| \| Control vs. AIA + vehicle \| -96.61 \| Yes \| **** \| \| Control vs. AIA + C-PC 2 mg/kg \| -45.91 \| Yes \| * \| \| Control vs. AIA + C-PC 4 mg/kg \| -51.49 \| Yes \| ** \| \| Control vs. AIA + C-PC 8 mg/kg \| -51.15 \| Yes \| * \| \| AIA + vehicle vs. AIA + C-PC 2 mg/kg \| 50.70 \| Yes \| ** \| \| AIA + vehicle vs. AIA + C-PC 4 mg/kg \| 45.12 \| Yes \| * \| \| AIA + vehicle vs. AIA + C-PC 8 mg/kg \| 45.46 \| Yes \| * \| \| AIA + C-PC 2 mg/kg vs. AIA + C-PC 4 mg/kg \| -5.582 \| No \| ns \| \| AIA + C-PC 2 mg/kg vs. AIA + C-PC 8 mg/kg \| -5.243 \| No \| ns \| \| AIA + C-PC 4 mg/kg vs. AIA + C-PC 8 mg/kg \| 0.3383 \| No \| ns \|   **MPO activity (arbitrary units)**   \| **One-way ANOVA** \| **SS** \| **DF** \| **MS** \| **F (DFn, DFd)** \| **P value** \| \| --- \| --- \| --- \| --- \| --- \| --- \| \| Treatment (between columns) \| 37.51 \| 4 \| 9.377 \| F (4, 69) = 21.87 \| P<0.0001 \| \| Residual (within columns) \| 29.58 \| 69 \| 0.4287 \|  \|  \| \| Total \| 67.09 \| 73 \|  \|  \|  \|  \| **Tukey’s multiple comparisons test** \| **Mean Diff.** \| **Significant?** \| **Summary** \| \| --- \| --- \| --- \| --- \| \| Control vs. AIA + vehicle \| -1.604 \| Yes \| **** \| \| Control vs. AIA + C-PC 2 mg/kg \| -0.3897 \| No \| ns \| \| Control vs. AIA + C-PC 4 mg/kg \| -0.3547 \| No \| ns \| \| Control vs. AIA + C-PC 8 mg/kg \| -0.3918 \| No \| ns \| \| AIA + vehicle vs. AIA + C-PC 2 mg/kg \| 1.214 \| Yes \| ** \| \| AIA + vehicle vs. AIA + C-PC 4 mg/kg \| 1.249 \| Yes \| *** \| \| AIA + vehicle vs. AIA + C-PC 8 mg/kg \| 1.212 \| Yes \| ** \| \| AIA + C-PC 2 mg/kg vs. AIA + C-PC 4 mg/kg \| 0.03503 \| No \| ns \| \| AIA + C-PC 2 mg/kg vs. AIA + C-PC 8 mg/kg \| -0.002015 \| No \| ns \| \| AIA + C-PC 4 mg/kg vs. AIA + C-PC 8 mg/kg \| -0.03705 \| No \| ns \|   **CXCL1 (pg/mL)**   \| **One-way ANOVA** \| **SS** \| **DF** \| **MS** \| **F (DFn, DFd)** \| **P value** \| \| --- \| --- \| --- \| --- \| --- \| --- \| \| Treatment (between columns) \| 28.25 \| 4 \| 7.061 \| F (4, 21) = 14.25 \| P<0.0001 \| \| Residual (within columns) \| 10.41 \| 21 \| 0.4956 \|  \|  \| \| Total \| 38.65 \| 25 \|  \|  \|  \|  \| **Tukey’s multiple comparisons test** \| **Mean Diff.** \| **Significant?** \| **Summary** \| \| --- \| --- \| --- \| --- \| \| Control vs. AIA + vehicle \| -3.172 \| Yes \| **** \| \| Control vs. AIA + C-PC 2 mg/kg \| -0.6312 \| No \| ns \| \| Control vs. AIA + C-PC 4 mg/kg \| -0.9328 \| No \| ns \| \| Control vs. AIA + C-PC 8 mg/kg \| -0.7961 \| No \| ns \| \| AIA + vehicle vs. AIA + C-PC 2 mg/kg \| 2.541 \| Yes \| **** \| \| AIA + vehicle vs. AIA + C-PC 4 mg/kg \| 2.239 \| Yes \| *** \| \| AIA + vehicle vs. AIA + C-PC 8 mg/kg \| 2.376 \| Yes \| *** \| \| AIA + C-PC 2 mg/kg vs. AIA + C-PC 4 mg/kg \| -0.3017 \| No \| ns \| \| AIA + C-PC 2 mg/kg vs. AIA + C-PC 8 mg/kg \| -0.1649 \| No \| ns \| \| AIA + C-PC 4 mg/kg vs. AIA + C-PC 8 mg/kg \| 0.1367 \| No \| ns \| |
| --- | --- | --- | --- | --- | --- | --- | --- | --- | --- | --- | --- | --- | --- | --- | --- | --- | --- | --- | --- | --- | --- | --- | --- | --- | --- | --- | --- | --- | --- | --- | --- | --- | --- | --- | --- | --- | --- | --- | --- | --- | --- | --- | --- | --- | --- | --- | --- | --- | --- | --- | --- | --- | --- | --- | --- | --- | --- | --- | --- | --- | --- | --- | --- | --- | --- | --- | --- | --- | --- | --- | --- | --- | --- | --- | --- | --- | --- | --- | --- | --- | --- | --- | --- | --- | --- | --- | --- | --- | --- | --- | --- | --- | --- | --- | --- | --- | --- | --- | --- | --- | --- | --- | --- | --- | --- | --- | --- | --- | --- | --- | --- | --- | --- | --- | --- | --- | --- | --- | --- | --- | --- | --- | --- | --- | --- | --- | --- | --- | --- | --- | --- | --- | --- | --- | --- | --- | --- | --- | --- | --- | --- | --- | --- | --- | --- | --- | --- | --- | --- | --- | --- | --- | --- | --- | --- | --- | --- | --- | --- | --- | --- | --- | --- | --- | --- | --- | --- | --- | --- | --- | --- | --- | --- | --- | --- | --- | --- | --- | --- | --- | --- | --- | --- | --- | --- | --- | --- | --- | --- | --- | --- | --- | --- | --- | --- | --- | --- | --- | --- | --- | --- | --- | --- | --- | --- | --- | --- | --- | --- | --- | --- | --- | --- | --- | --- | --- | --- | --- | --- | --- | --- | --- | --- | --- | --- | --- | --- | --- | --- | --- | --- | --- | --- | --- | --- | --- | --- | --- | --- | --- | --- | --- | --- | --- | --- | --- | --- | --- | --- | --- | --- | --- | --- | --- | --- | --- | --- | --- | --- | --- | --- | --- | --- | --- | --- | --- | --- | --- | --- | --- | --- | --- | --- |
| **Figure 2.** | **Individual data of biodistribution at 24h post intraperitoneal administration, n=4**   \| Sample \| Units \| 1 \| 2 \| 3 \| 4 \| \| --- \| --- \| --- \| --- \| --- \| --- \| \| Liver \| %D/g \| 0.14 \| 0.16 \| 0.18 \| 0.12 \| \| Spleen \| %D/g \| 0.13 \| 0.14 \| 0.14 \| 0.08 \| \| Kydneys \| %D/g \| 0.58 \| 0.53 \| 0.55 \| 0.11 \| \| Heart \| %D/g \| 0.070 \| 0.080 \| 0.08 \| 0.07 \| \| Lungs \| %D/g \| 0.110 \| 0.130 \| 0.18 \| 0.13 \| \| Brain \| %D/g \| 0.02 \| 0.01 \| 0.01 \| 0.01 \| \| Muscle \| %D/g \| 0.02 \| 0.03 \| 0.04 \| 0.04 \| \| Adipocytes \| %D/g \| 0.33 \| 0.07 \| 0.10 \| 0.02 \| \| Stomach \| %D \| 1.67 \| 2.00 \| 3.23 \| 2.46 \| \| Small intestine \| %D \| 0.66 \| 1.96 \| 1.20 \| 0.84 \| \| Large intestine \| %D \| 0.77 \| 0.87 \| 0.92 \| 3.50 \|   **Individual data of biodistribution at 24h post intravenous administration, n=5**   \| Sample \| Units \| 1 \| 2 \| 3 \| 4 \| 5 \| \| --- \| --- \| --- \| --- \| --- \| --- \| --- \| \| Liver \| %D/g \| 0.09 \| 0.07 \| 0.09 \| 0.16 \| 0.15 \| \| Spleen \| %D/g \| 0.10 \| 0.08 \| 0.06 \| 0.08 \| 0.08 \| \| Kidneys \| %D/g \| 0.54 \| 0.67 \| 0.47 \| 0.57 \| 0.46 \| \| Heart \| %D/g \| 0.03 \| 0.04 \| 0.04 \| 0.05 \| 0.04 \| \| Lungs \| %D/g \| 0.06 \| 0.07 \| 0.06 \| 0.11 \| 0.07 \| \| Brain \| %D/g \| 0.01 \| 0.01 \| 0.01 \| 0.01 \| 0.01 \| \| Muscle \| %D/g \| 0.02 \| 0.02 \| 0.01 \| 0.03 \| 0.02 \| \| Adipocytes \| %D/g \| 0.03 \| 0.02 \| 0.02 \| 0.06 \| 0.04 \| \| Stomach \| %D \| 0.56 \| 0.95 \| 0.64 \| 2.13 \| 0.87 \| \| Small intestine \| %D \| 0.43 \| 0.52 \| 0.34 \| 0.51 \| 0.72 \| \| Large Intestine \| %D \| 0.23 \| 0.80 \| 0.66 \| 0.60 \| 0.69 \|   **Individual data of biodistribution at 24h post nasal administration, n=2**   \| Sample \| Units \| 1 \| 2 \| 3 \| 4.00 \| \| --- \| --- \| --- \| --- \| --- \| --- \| \| Liver* \| %D/g \| 0.04 \| 0.04 \| 0.04 \| 0.05 \| \| Spleen \| %D/g \| 0.03 \| 0.03 \|  \| \| \| Kidneys** \| %D/g \| 0.05 \| 0.05 \| 0.05 \| 0.05 \| \| Lymph nodes \| %D/g \| 0.003 \| 0.002 \|  \| \| \| Brain \| %D/g \| 0.004 \| 0.002 \| \| Stomach \| %D \| 1.01 \| 0.18 \| \| Small intestine \| %D \| 0.29 \| 0.56 \| \| Large intestine \| %D \| 0.29 \| 0.23 \| \| Nose \| %D \| 0.08 \| 0.07 \| \| * two pieces per animals; ** left and right kidneys measured separately \| \| \| \| \| \|   **Individual data of biodistribution at 24h post oral administration, n=2**   \| Sample \| Units \| 1.00 \| 2.00 \| 3 \| 4.00 \| \| --- \| --- \| --- \| --- \| --- \| --- \| \| Liver* \| %D/g \| 0.37 \| 2.18 \| 0.33 \| 2.17 \| \| Spleen \| %D/g \| 0.23 \| 1.86 \|  \| \| \| Kydneys** \| %D/g \| 0.46 \| 2.34 \| 0.51 \| 2.35 \| \| Brain \| %D/g \| 0.05 \| 0.30 \|  \| \| \| Stomach \| %D \| 15.20 \| 1.03 \| \| Small intestine \| %D \| 5.90 \| 4.40 \| \| Large intestine \| %D \| 27.80 \| 22.40 \| \| * two pieces per animals; ** left and right kidneys measured separately \| \| \| \| \| \| |
| **Figure 3.**  **Figure 4.** | **Intensity of hypernociception (Δ withdraw threshold, g)**   \| **One-way ANOVA** \| **SS** \| **DF** \| **MS** \| **F (DFn, DFd)** \| **P value** \| \| --- \| --- \| --- \| --- \| --- \| --- \| \| Treatment (between columns) \| 109.6 \| 3 \| 36.53 \| F (3, 34) = 98.50 \| P<0.0001 \| \| Residual (within columns) \| 12.61 \| 34 \| 0.3709 \|  \|  \| \| Total \| 122.2 \| 37 \|  \|  \|  \|  \| **Tukey’s multiple comparisons test** \| **Mean Diff.** \| **Significant?** \| **Summary** \| \| --- \| --- \| --- \| --- \| \| Control vs. AIA + vehicle \| -4.076 \| Yes \| **** \| \| Control vs. AIA + PCB 0.1 mg/kg \| -1.017 \| Yes \| ** \| \| Control vs. AIA + PCB 1 mg/kg \| -0.3850 \| No \| ns \| \| AIA + vehicle vs. AIA + PCB 0.1 mg/kg \| 3.059 \| Yes \| **** \| \| AIA + vehicle vs. AIA + PCB 1 mg/kg \| 3.691 \| Yes \| **** \| \| AIA + PCB 0.1 mg/kg vs. AIA + PCB 1 mg/kg \| 0.6317 \| No \| ns \|   **# neutrophils x 10^4^ (Per synovial cavity)**   \| **One-way ANOVA** \| **SS** \| **DF** \| **MS** \| **F (DFn, DFd)** \| **P value** \| \| --- \| --- \| --- \| --- \| --- \| --- \| \| Treatment (between columns) \| 80281 \| 3 \| 26760 \| F (3, 48) = 41.00 \| P<0.0001 \| \| Residual (within columns) \| 31326 \| 48 \| 652.6 \|  \|  \| \| Total \| 111607 \| 51 \|  \|  \|  \|  \| **Tukey’s multiple comparisons test** \| **Mean Diff.** \| **Significant?** \| **Summary** \| \| --- \| --- \| --- \| --- \| \| Control vs. AIA + vehicle \| -96.48 \| Yes \| **** \| \| Control vs. AIA + PCB 0.1 mg/kg \| -46.79 \| Yes \| *** \| \| Control vs. AIA + PCB 1 mg/kg \| -21.10 \| No \| ns \| \| AIA + vehicle vs. AIA + PCB 0.1 mg/kg \| 49.69 \| Yes \| **** \| \| AIA + vehicle vs. AIA + PCB 1 mg/kg \| 75.38 \| Yes \| **** \| \| AIA + PCB 0.1 mg/kg vs. AIA + PCB 1 mg/kg \| 25.69 \| No \| ns \|   **MPO activity (arbitrary units)**   \| **One-way ANOVA** \| **SS** \| **DF** \| **MS** \| **F (DFn, DFd)** \| **P value** \| \| --- \| --- \| --- \| --- \| --- \| --- \| \| Treatment (between columns) \| 42.00 \| 3 \| 14.00 \| F (3, 83) = 39.78 \| P<0.0001 \| \| Residual (within columns) \| 29.21 \| 83 \| 0.3519 \|  \|  \| \| Total \| 71.22 \| 86 \|  \|  \|  \|  \| **Tukey’s multiple comparisons test** \| **Mean Diff.** \| **Significant?** \| **Summary** \| \| --- \| --- \| --- \| --- \| \| Control vs. AIA + vehicle \| -1.604 \| Yes \| **** \| \| Control vs. AIA + PCB 0.1 mg/kg \| -0.1862 \| No \| ns \| \| Control vs. AIA + PCB 1 mg/kg \| -0.1286 \| No \| ns \| \| AIA + vehicle vs. AIA + PCB 0.1 mg/kg \| 1.418 \| Yes \| **** \| \| AIA + vehicle vs. AIA + PCB 1 mg/kg \| 1.476 \| Yes \| **** \| \| AIA + PCB 0.1 mg/kg vs. AIA + PCB 1 mg/kg \| 0.05766 \| No \| ns \|   **T-bet mRNA relative expression (fold change)**   \| **One-way ANOVA** \| **SS** \| **DF** \| **MS** \| **F (DFn, DFd)** \| **P value** \| \| --- \| --- \| --- \| --- \| --- \| --- \| \| Treatment (between columns) \| 1788 \| 3 \| 596.0 \| F (3, 18) = 3.083 \| P=0.0536 \| \| Residual (within columns) \| 3480 \| 18 \| 193.3 \|  \|  \| \| Total \| 5268 \| 21 \|  \|  \|  \|  \| **Tukey’s multiple comparisons test** \| **Mean Diff.** \| **Significant?** \| **Summary** \| \| --- \| --- \| --- \| --- \| \| Control vs. AIA + vehicle \| -17.69 \| No \| ns \| \| Control vs. AIA + PCB 0.1 mg/kg \| -4.990 \| No \| ns \| \| Control vs. AIA + PCB 1 mg/kg \| 6.831 \| No \| ns \| \| AIA + vehicle vs. AIA + PCB 0.1 mg/kg \| 12.70 \| No \| ns \| \| AIA + vehicle vs. AIA + PCB 1 mg/kg \| 24.52 \| Yes \| * \| \| AIA + PCB 0.1 mg/kg vs. AIA + PCB 1 mg/kg \| 11.82 \| No \| ns \|   **RORγ mRNA relative expression (fold change)**   \| **One-way ANOVA** \| **SS** \| **DF** \| **MS** \| **F (DFn, DFd)** \| **P value** \| \| --- \| --- \| --- \| --- \| --- \| --- \| \| Treatment (between columns) \| 13960 \| 3 \| 4653 \| F (3, 17) = 3.633 \| P=0.0343 \| \| Residual (within columns) \| 21772 \| 17 \| 1281 \|  \|  \| \| Total \| 35732 \| 20 \|  \|  \|  \|  \| **Tukey’s multiple comparisons test** \| **Mean Diff.** \| **Significant?** \| **Summary** \| \| --- \| --- \| --- \| --- \| \| Control vs. AIA + vehicle \| -51.38 \| No \| ns \| \| Control vs. AIA + PCB 0.1 mg/kg \| 5.890 \| No \| ns \| \| Control vs. AIA + PCB 1 mg/kg \| 25.60 \| No \| ns \| \| AIA + vehicle vs. AIA + PCB 0.1 mg/kg \| 57.27 \| No \| ns \| \| AIA + vehicle vs. AIA + PCB 1 mg/kg \| 76.98 \| Yes \| * \| \| AIA + PCB 0.1 mg/kg vs. AIA + PCB 1 mg/kg \| 19.71 \| No \| ns \|   **IFN-γ mRNA relative expression (fold change)**   \| **One-way ANOVA** \| **SS** \| **DF** \| **MS** \| **F (DFn, DFd)** \| **P value** \| \| --- \| --- \| --- \| --- \| --- \| --- \| \| Treatment (between columns) \| 451.0 \| 3 \| 150.3 \| F (3, 16) = 3.842 \| P=0.0302 \| \| Residual (within columns) \| 626.1 \| 16 \| 39.13 \|  \|  \| \| Total \| 1077 \| 19 \|  \|  \|  \|  \| **Tukey’s multiple comparisons test** \| **Mean Diff.** \| **Significant?** \| **Summary** \| \| --- \| --- \| --- \| --- \| \| Control vs. AIA + vehicle \| -11.13 \| No \| ns \| \| Control vs. AIA + PCB 0.1 mg/kg \| -4.797 \| No \| ns \| \| Control vs. AIA + PCB 1 mg/kg \| 1.076 \| No \| ns \| \| AIA + vehicle vs. AIA + PCB 0.1 mg/kg \| 6.337 \| No \| ns \| \| AIA + vehicle vs. AIA + PCB 1 mg/kg \| 12.21 \| Yes \| * \| \| AIA + PCB 0.1 mg/kg vs. AIA + PCB 1 mg/kg \| 5.872 \| No \| ns \|   **IFN-γ (pg/mL)**   \| **One-way ANOVA** \| **SS** \| **DF** \| **MS** \| **F (DFn, DFd)** \| **P value** \| \| --- \| --- \| --- \| --- \| --- \| --- \| \| Treatment (between columns) \| 0.1027 \| 3 \| 0.03423 \| F (3, 16) = 8.343 \| P=0.0014 \| \| Residual (within columns) \| 0.06564 \| 16 \| 0.004103 \|  \|  \| \| Total \| 0.1683 \| 19 \|  \|  \|  \|  \| **Tukey’s multiple comparisons test** \| **Mean Diff.** \| **Significant?** \| **Summary** \| \| --- \| --- \| --- \| --- \| \| Control vs. AIA + vehicle \| -0.1860 \| Yes \| ** \| \| Control vs. AIA + PCB 0.1 mg/kg \| -0.1180 \| Yes \| * \| \| Control vs. AIA + PCB 1 mg/kg \| -0.04000 \| No \| ns \| \| AIA + vehicle vs. AIA + PCB 0.1 mg/kg \| 0.06800 \| No \| ns \| \| AIA + vehicle vs. AIA + PCB 1 mg/kg \| 0.1460 \| Yes \| * \| \| AIA + PCB 0.1 mg/kg vs. AIA + PCB 1 mg/kg \| 0.07800 \| No \| ns \|   **TNF-α (pg/mL)**   \| **Kruskal-Wallis test** \|  \| \| --- \| --- \| \| P value \| 0.0258 \| \| Exact or approximate P value? \| Approximate \| \| P value summary \| * \| \| Do the medians vary signif. (P < 0.05)? \| Yes \| \| Number of groups \| 4 \| \| Kruskal-Wallis statistic \| 9.275 \|  \| **Dunn’s multiple comparisons test** \| **Mean Diff.** \| **Significant?** \| **Summary** \| \| --- \| --- \| --- \| --- \| \| Control vs. AIA + vehicle \| -10.87 \| Yes \| * \| \| Control vs. AIA + PCB 0.1 mg/kg \| -7.700 \| No \| ns \| \| Control vs. AIA + PCB 1 mg/kg \| -2.800 \| No \| ns \| \| AIA + vehicle vs. AIA + PCB 0.1 mg/kg \| 3.167 \| No \| ns \| \| AIA + vehicle vs. AIA + PCB 1 mg/kg \| 8.067 \| No \| ns \| \| AIA + PCB 0.1 mg/kg vs. AIA + PCB 1 mg/kg \| 4.900 \| No \| ns \|   **IL-17A (pg/mL)**   \| **Kruskal-Wallis test** \|  \| \| --- \| --- \| \| P value \| 0.0026 \| \| Exact or approximate P value? \| Approximate \| \| P value summary \| ** \| \| Do the medians vary signif. (P < 0.05)? \| Yes \| \| Number of groups \| 4 \| \| Kruskal-Wallis statistic \| 14.21 \|  \| **Dunn’s multiple comparisons test** \| **Mean Diff.** \| **Significant?** \| **Summary** \| \| --- \| --- \| --- \| --- \| \| Control vs. AIA + vehicle \| -11.57 \| Yes \| * \| \| Control vs. AIA + PCB 0.1 mg/kg \| -9.650 \| No \| ns \| \| Control vs. AIA + PCB 1 mg/kg \| -0.5000 \| No \| ns \| \| AIA + vehicle vs. AIA + PCB 0.1 mg/kg \| 1.917 \| No \| ns \| \| AIA + vehicle vs. AIA + PCB 1 mg/kg \| 11.07 \| Yes \| * \| \| AIA + PCB 0.1 mg/kg vs. AIA + PCB 1 mg/kg \| 9.150 \| No \| ns \|   **IL-4 (pg/mL)**   \| **One-way ANOVA** \| **SS** \| **DF** \| **MS** \| **F (DFn, DFd)** \| **P value** \| \| --- \| --- \| --- \| --- \| --- \| --- \| \| Treatment (between columns) \| 0.09769 \| 3 \| 0.03256 \| F (3, 18) = 7.094 \| P=0.0024 \| \| Residual (within columns) \| 0.08263 \| 18 \| 0.004591 \|  \|  \| \| Total \| 0.1803 \| 21 \|  \|  \|  \|  \| **Tukey’s multiple comparisons test** \| **Mean Diff.** \| **Significant?** \| **Summary** \| \| --- \| --- \| --- \| --- \| \| Control vs. AIA + vehicle \| -0.1690 \| Yes \| ** \| \| Control vs. AIA + PCB 0.1 mg/kg \| -0.03233 \| No \| ns \| \| Control vs. AIA + PCB 1 mg/kg \| -0.03200 \| No \| ns \| \| AIA + vehicle vs. AIA + PCB 0.1 mg/kg \| 0.1367 \| Yes \| * \| \| AIA + vehicle vs. AIA + PCB 1 mg/kg \| 0.1370 \| Yes \| * \| \| AIA + PCB 0.1 mg/kg vs. AIA + PCB 1 mg/kg \| 0.0003333 \| No \| ns \| |
| **Figure 5.** | **Arthritis index**   \| **One-way ANOVA** \| **SS** \| **DF** \| **MS** \| **F (DFn, DFd)** \| **P value** \| \| --- \| --- \| --- \| --- \| --- \| --- \| \| Treatment (between columns) \| 32.29 \| 2 \| 16.15 \| F (2, 11) = 21.64 \| P=0.0002 \| \| Residual (within columns) \| 8.208 \| 11 \| 0.7462 \|  \|  \| \| Total \| 40.50 \| 13 \|  \|  \|  \|  \| **Tukey’s multiple comparisons test** \| **Mean Diff.** \| **Significant?** \| **Summary** \| \| --- \| --- \| --- \| --- \| \| Control vs. AIA + vehicle \| -4.000 \| Yes \| *** \| \| Control vs. AIA + PCB 1 mg/kg \| -1.708 \| Yes \| * \| \| AIA + vehicle vs. AIA + PCB 1 mg/kg \| 2.292 \| Yes \| ** \| |
