# Supplementary figures and images for: The effects of Phycocyanobilin on experimental arthritis involve the reduction in nociception and synovial neutrophil infiltration, inhibition of cytokine production, and modulation of the neuronal proteome
Source: Front Immunol. 2023 Oct 23;14:1227268. doi: 10.3389/fimmu.2023.1227268 (PMC10627171; doi:10.3389/fimmu.2023.1227268)

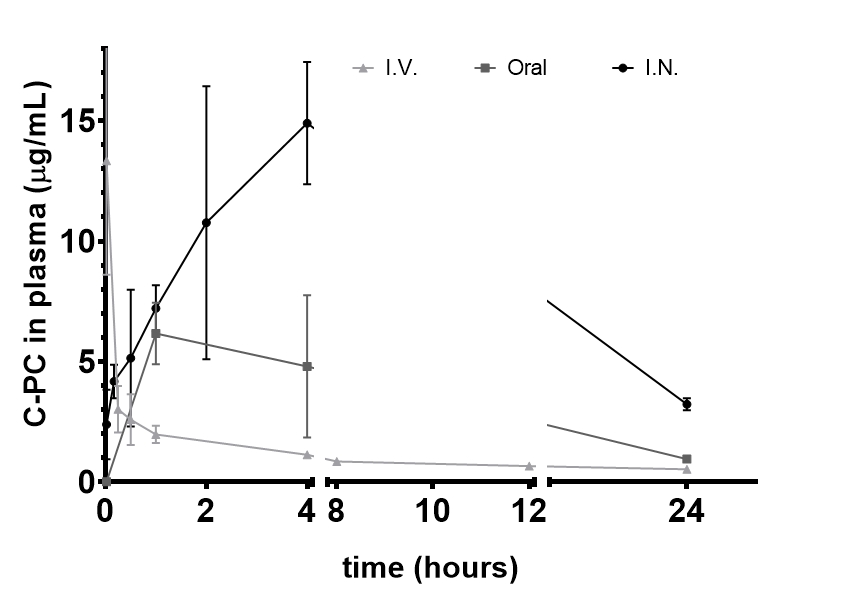

Supplement: Supplementary Figure 1 — Plasma concentration of C-PC after oral (squares), i.n. (circles) and i.v. (triangle) administrations at doses as described in the Material and Method section. [file Image_1.jpeg]
