# Supplementary material for: The effects of Phycocyanobilin on experimental arthritis involve the reduction in nociception and synovial neutrophil infiltration, inhibition of cytokine production, and modulation of the neuronal proteome
Source: Front Immunol. 2023 Oct 23;14:1227268. doi: 10.3389/fimmu.2023.1227268 (PMC10627171; doi:10.3389/fimmu.2023.1227268)
Supplement: Supplementary Figure 2 — (A) PCA analysis of protein cell extracts after PCB treatment versus control cells demonstrates how both groups are different; red and blue squares correspond to PCB-treated and non-treated replicates, respectively. (B) Volcano plot showing the proteins differentially modulated (p<0.05) after PCB treatment in comparison to control cells. (C) Hierarchical clustering of proteins differentially expressed by PCB treatment compared to control cells (three replicates per each condition). Up- and down-regulated genes are in blue and pink colors, respectively. [file Image_2.pdf]

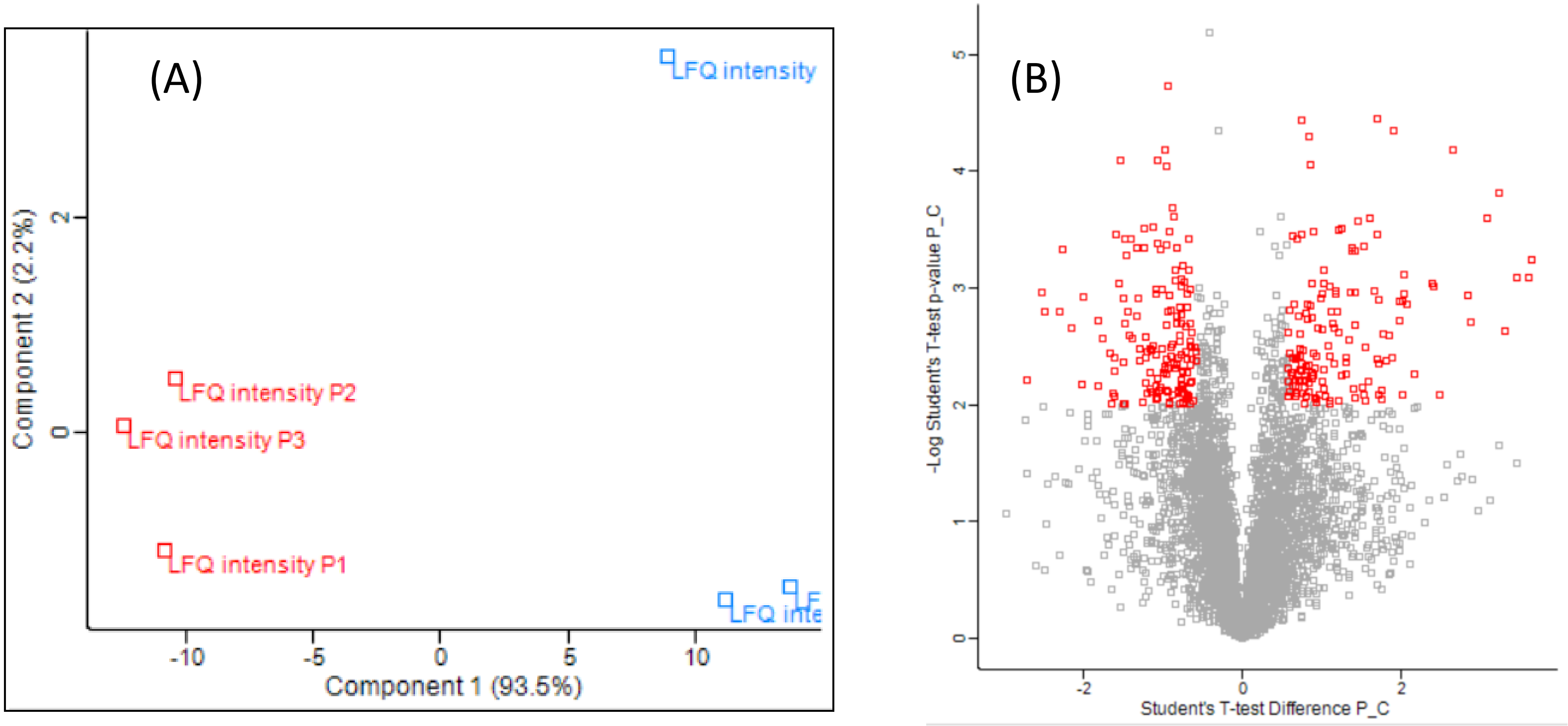

(A) PCA analysis of protein cell extracts after PCB treatment versus control cells that demonstrate how different are both groups; red and blue squares correspond to PCB-treated and non-treated replicates, respectively. (B) Volcano plot showing the proteins differentialy expressed ($p<0.05$) after PCB treatment in comparison to control cells. 156 proteins were down-regulated while 146 proteins were up-regulated with the treatment. Abscissas are $-\log_{10}$ (p-value) versus $\log_2$ (fold-change).

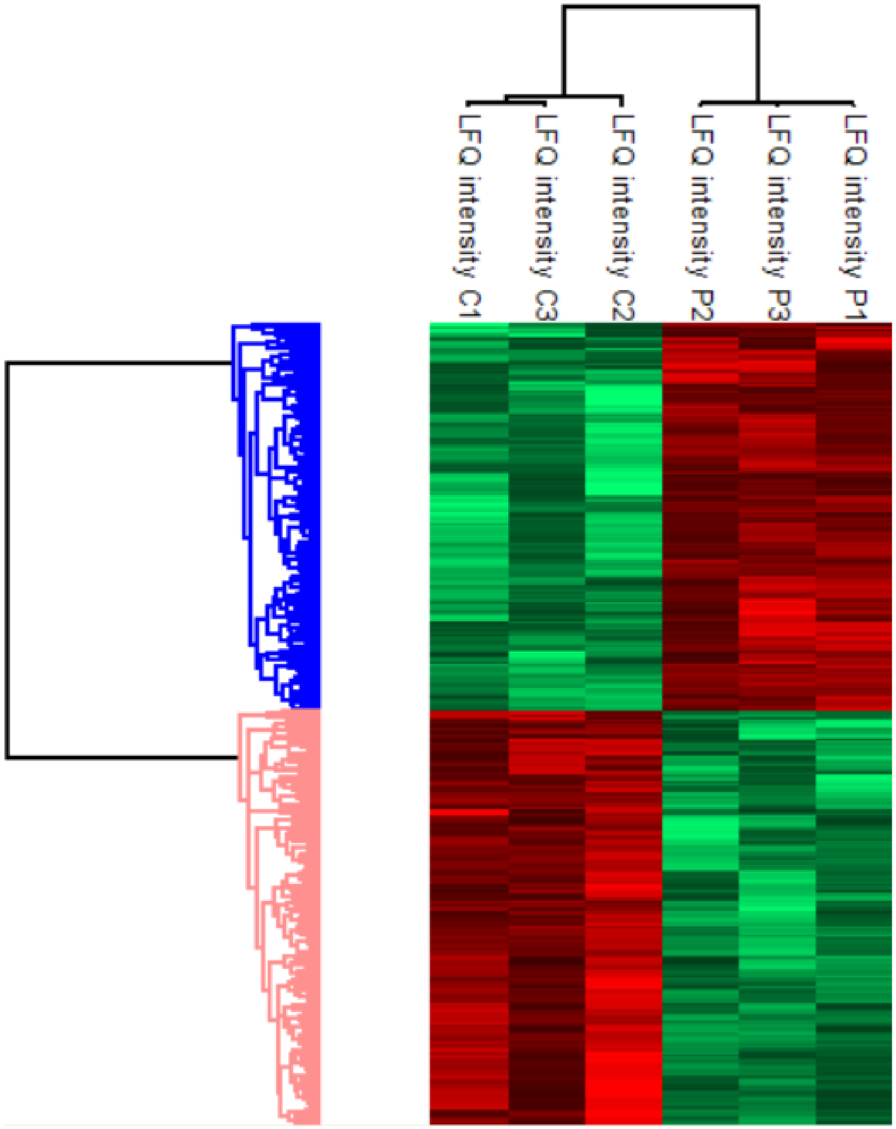

(C) Hierarchical clustering of proteins differentially expressed by PCB treatment compared to control cells (three replicates per each condition). Up- and down-regulated genes are in blue and pink colors, respectively.
