# Supplementary material for: The effects of Phycocyanobilin on experimental arthritis involve the reduction in nociception and synovial neutrophil infiltration, inhibition of cytokine production, and modulation of the neuronal proteome
Source: Front Immunol. 2023 Oct 23;14:1227268. doi: 10.3389/fimmu.2023.1227268 (PMC10627171; doi:10.3389/fimmu.2023.1227268)
Supplement: Supplementary file 7 [file Presentation_1.pptx]

## Slide 1
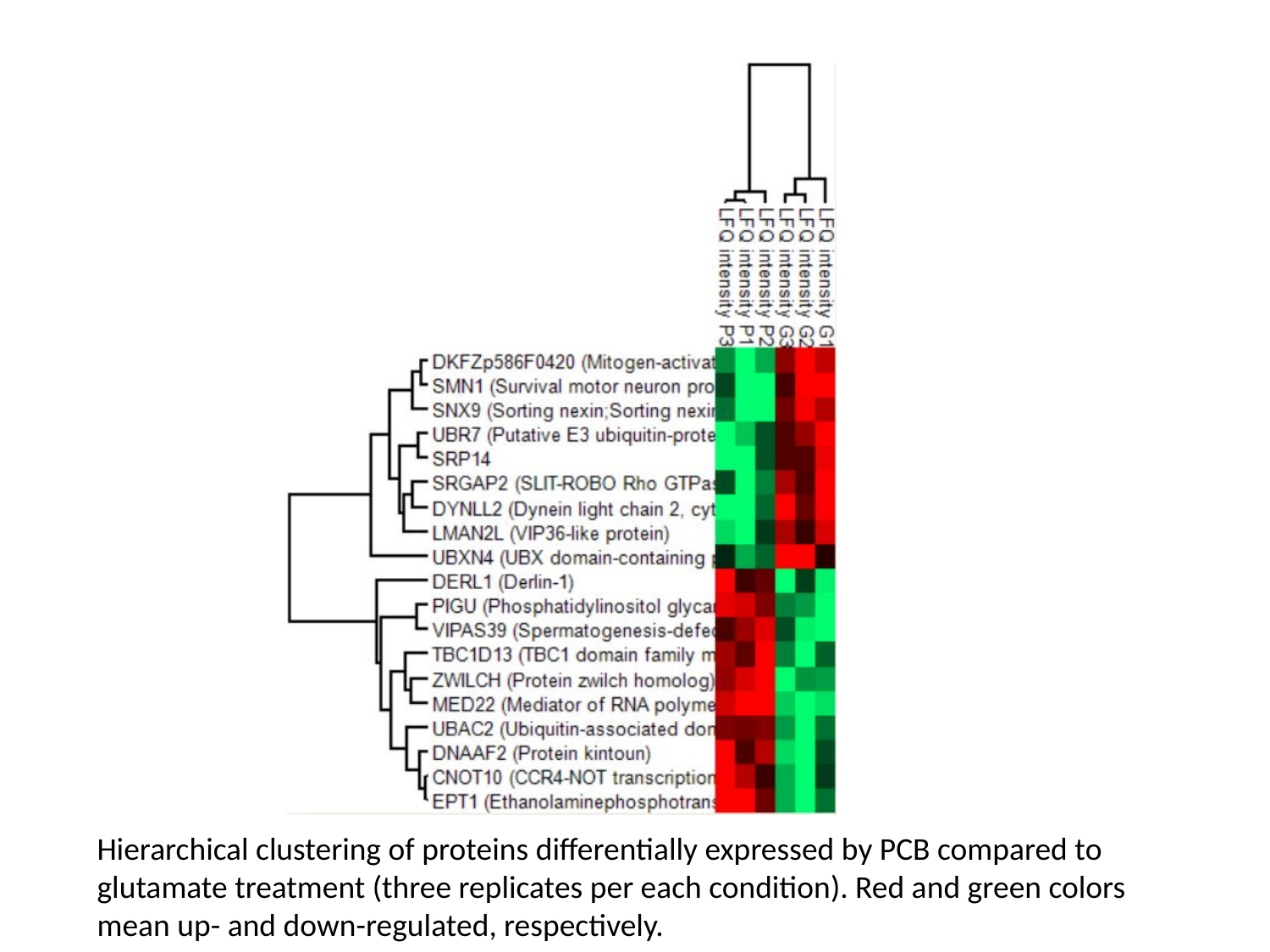

Hierarchical clustering of proteins differentially expressed by PCB compared to glutamate treatment (three replicates per each condition). Red and green colors mean up- and down-regulated, respectively.

## Slide 2
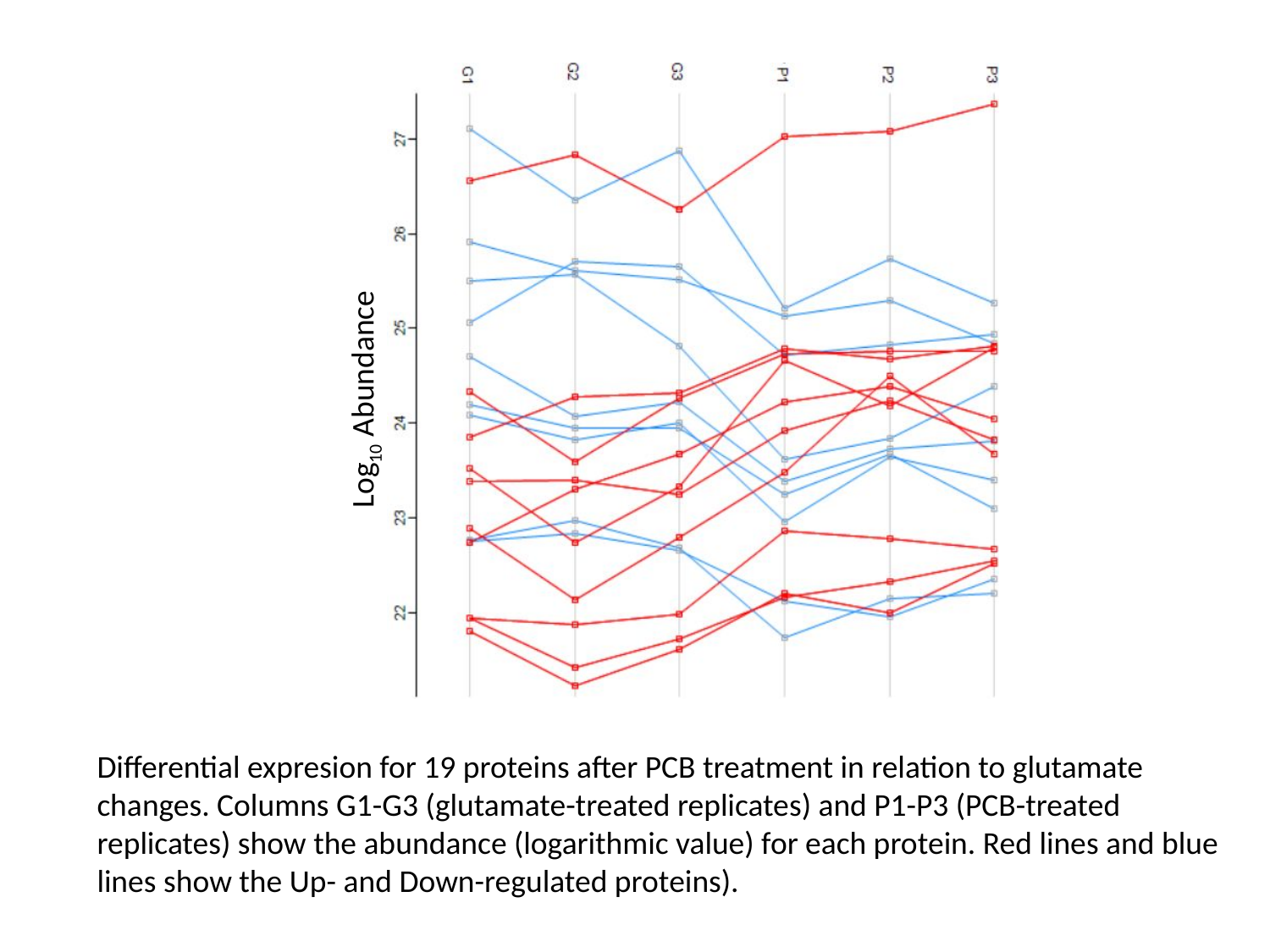

Log10 Abundance
Differential expresion for 19 proteins after PCB treatment in relation to glutamate changes. Columns G1-G3 (glutamate-treated replicates) and P1-P3 (PCB-treated replicates) show the abundance (logarithmic value) for each protein. Red lines and blue lines show the Up- and Down-regulated proteins).

## Slide 3
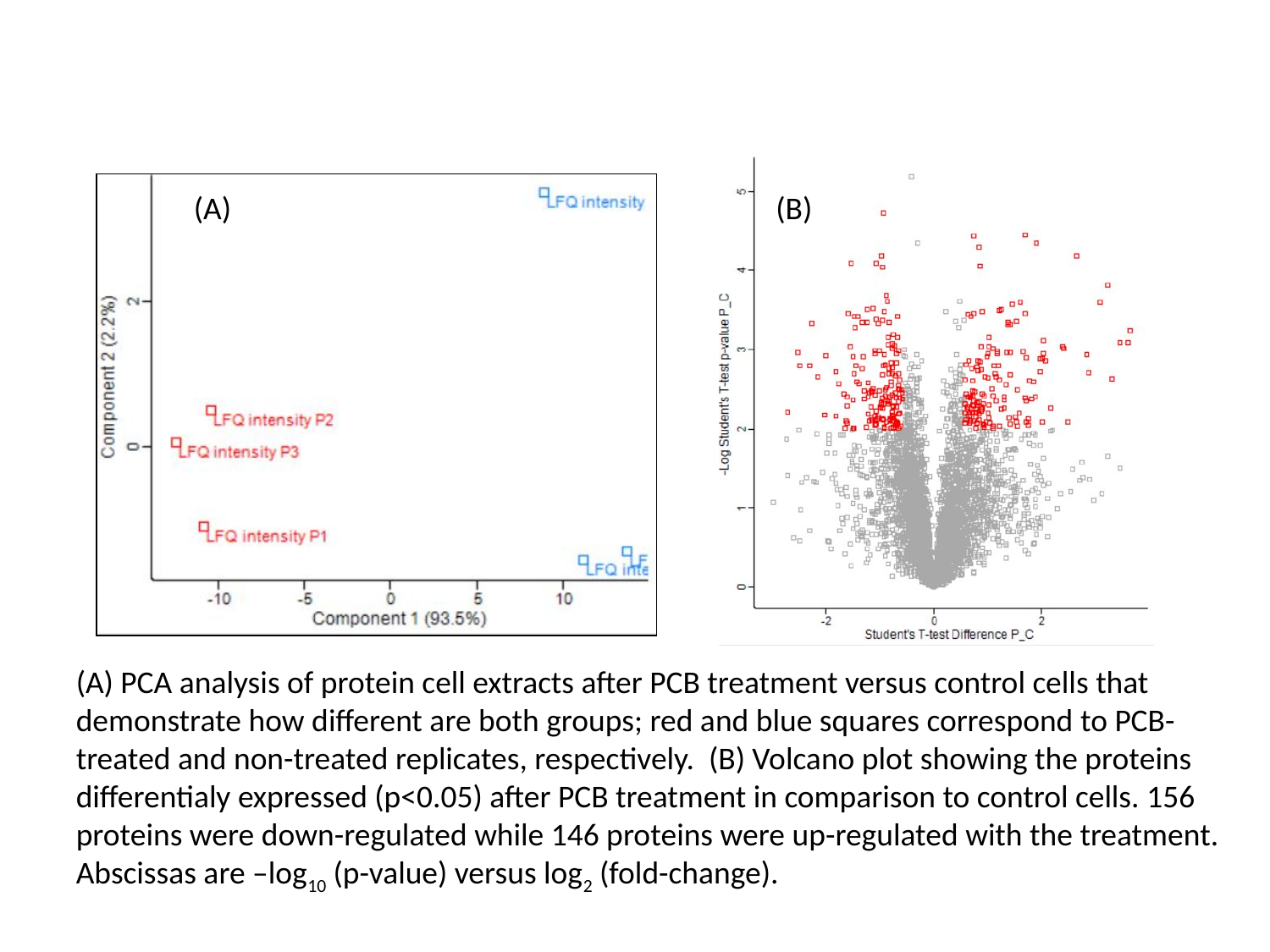

(A)
(B)
(A) PCA analysis of protein cell extracts after PCB treatment versus control cells that demonstrate how different are both groups; red and blue squares correspond to PCB-treated and non-treated replicates, respectively. (B) Volcano plot showing the proteins differentialy expressed (p<0.05) after PCB treatment in comparison to control cells. 156 proteins were down-regulated while 146 proteins were up-regulated with the treatment. Abscissas are –log10 (p-value) versus log2 (fold-change).

## Slide 4
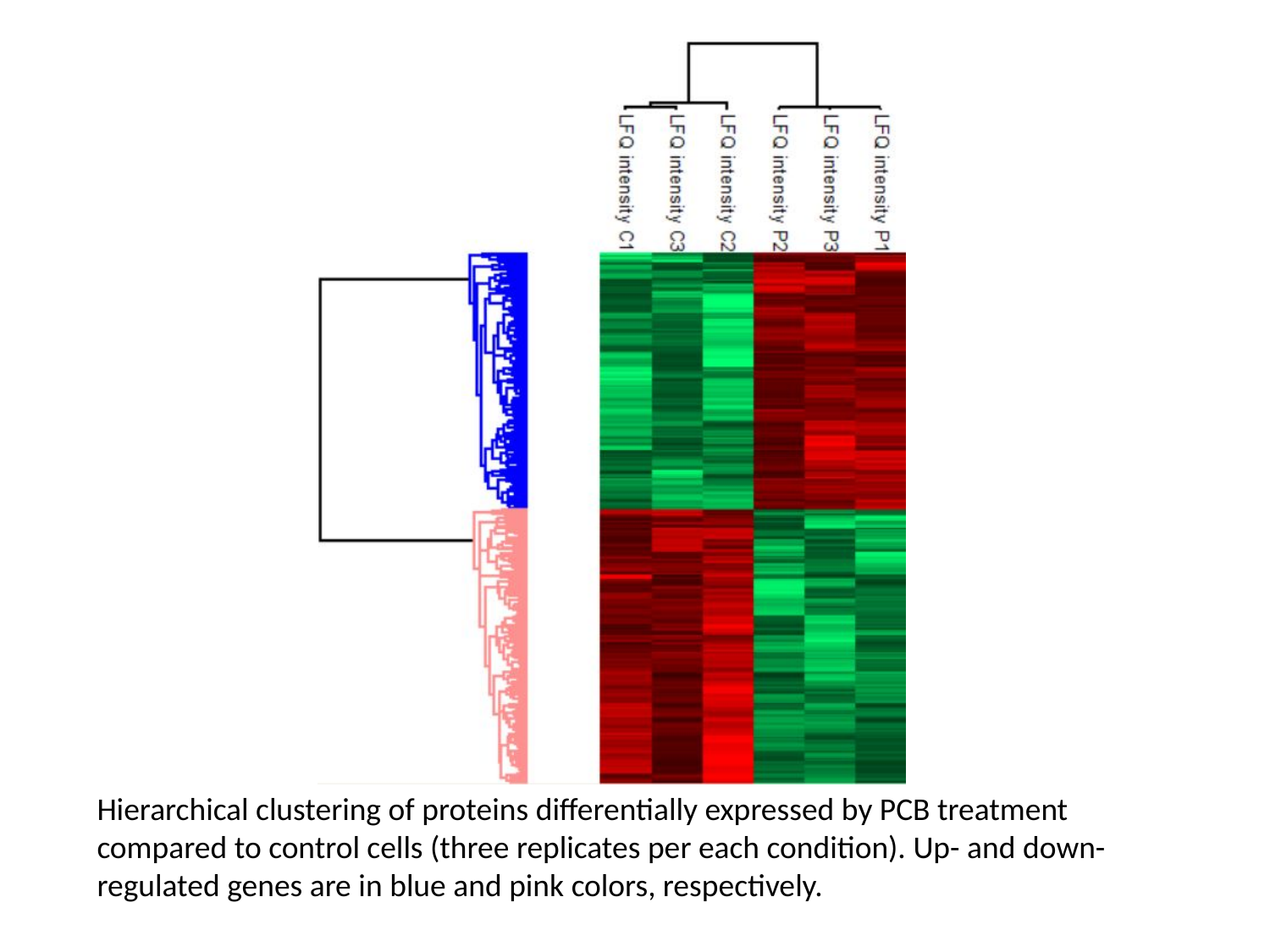

Hierarchical clustering of proteins differentially expressed by PCB treatment compared to control cells (three replicates per each condition). Up- and down-regulated genes are in blue and pink colors, respectively.
